# Compositionality and systematicity emerge from iterated learning in deep linear networks

**DOI:** 10.1073/pnas.2509739123

**Published:** 2026-05-05

**Authors:** Devon Jarvis, Richard Klein, Benjamin Rosman, Andrew M. Saxe

**Affiliations:** ^a^School of Computer Science and Applied Mathematics, University of the Witwatersrand, Johannesburg 2000, South Africa; ^b^Machine Intelligence and Neural Discovery Institute, University of the Witwatersrand, Johannesburg 2000, South Africa; ^c^Gatsby Computational Neuroscience Unit, University College London, London W1T 4JG, United Kingdom; ^d^Sainsbury Wellcome Centre, University College London, London W1T 4JG, United Kingdom

**Keywords:** systematic generalization, iterated Learning, linear neural networks

## Abstract

Humans have a remarkable ability to systematically generalize—reasoning about new situations by combining aspects of previous experiences. Language provides one of the primary examples of this ability and modern machine learning has drawn much inspiration from linguistics. A recent example is iterated learning, a procedure where generations of networks learn from the output of earlier learners. The result is a refinement of the network’s “language” or output labels for given inputs toward compositional structure. Here we theoretically study the emergence of compositional language, and the ability of simple neural networks to leverage this compositionality to systematically generalize. We build on prior theoretical work on linear networks, which mathematically define systematic generalization, by a) applying the analysis of shallow and deep linear network to the iterated learning procedure by deriving exact dynamics of learning over generations; b) refining the definition of systematicity to understand the benefits and limitations of iterated learning. We find that iterated learning does facilitate systematic generalization over standard training paradigms by uncovering compositional substructure in the output labels. Our results confirm a long standing conjecture: that multiple generations of iterated learning are required for compositional structure to emerge, which can outperform a single generation network trained with optimal early-stopping. However, for the network to treat the input systematically and ignore features which do not generalize, the network must be trained on an extremely large dataset. Hence, we define “weak systematic generalization” to explain this emergent systematicity from scale.

## Significance

Children rapidly acquire an ability for language during early development. One theory, called iterated learning, posits that language evolves over generations to become more structured. This structure can then be exploited by learners through systematic generalization, where past experiences are combined to handle new situations, contributing to this rapid development of language in children. We study the neural basis of systematic generalization and its implications for learning structured language. We find that iterated learning refines language but requires deep neural architectures to be effective. We highlight the limitations of iterated learning in that it cannot uncover regularity on the input features. To mitigate this, we show that networks tend toward systematicity as the vocabulary grows, showing emergent systematicity from scale.

Humans frequently display the ability to systematically generalize, that is, to leverage specific learning experiences in diverse new settings (1). For instance, exploiting the approximate compositionality of natural language, humans can combine a finite set of words and other morphemes into a near-infinite set of words and sentences which convey a near-infinite set of meanings. Someone who understands “brown dog” and “black cat” also likely understands “brown cat,” to take one example from Szabo (2). The result is that a human’s ability to communicate about situations or phenomena extends far beyond their ability to directly experience and learn all examples (3).

In this work, we study both sides of the above example: the source of the compositional nature of natural language and the properties of human learning which enable humans to exploit this compositionality. It has been proposed that the compositional nature of language results from dual pressures requiring it to be easily learnable but also expressive enough to be useful for communicating a wide range of concepts (4, 5). The iterated learning (IL) process provides a computational and mathematical framework for studying this cultural transmission and evolution of language (6, 7). IL is a process where a sequence of learners observe and learn a behavior from another learner who previously learned it through observation (8). Importantly, there is no explicit pressure or direction on how the knowledge changes when passed from learner to learner, as the only goal of one individual learner is to successfully reproduce the behavior (9). Yet, multiple models have shown that the behavior becomes easier to learn as the sequence of learners continues. Other works have also shown that as the size of communities grows, resulting in more behaviors to simultaneously be learned by each learner, the convergence toward easily reproduced behavior is sped up (10) and can even occur in a single generation (11). We do not consider this mechanism explicitly, although we draw comparisons to this mechanism when we consider dataset size later in the work.

A common application of the IL process to language sees generations of learners train briefly on a language produced by their parent, and then generate a new language for their child (5, 12–15). Language in this case is defined by the mapping between some object or thing which needs to be expressed (which we refer to as “meanings”) to the appropriate words or morphemes (referred to as “signals”) to convey those meanings. The result is that the language becomes simpler over generations as meaning is expressed using more reusable signals. However, in the absence of an environmental pressure to distinguish between meanings, the language will become overly simplified (degenerate) as signals are reused too frequently to describe overly broad groupings of objects or meanings. With a sufficient pressure to enforce expressivity, the language will instead simplify to signals which describe properties of objects or meanings and can be reused for multiple meanings with different combinations of properties. Such “compositional” reuse of signals supports both ease of learning (16) and the description of many meanings. Thus, IL can refine a language toward compositional structure while the irregular patterns in the language are forgotten (6) if there is sufficient incentive for the learner to learn to describe multiple meanings (9).

Kirby et al. (9) presents a direct evaluation of the IL algorithm with human learners. In their experiments, participants are tasked with learning a novel “alien” language which aims to describe a set of visual scenes depicting moving colored objects. The language in this case is the pairing of a vocabulary of words (the signals) uniquely labeling each scene (the meaning to be conveyed), where each word begins as an unstructured string of letters. It was found that within a few generations the language stabilized to a subset of highly reused strings. For example, all objects that moved horizontally were called “tuge” and all square objects that bounced were called “tupim.” These emergent regularities then support easy generalization. Even if a participant had not seen all versions of objects, colors, or movements, one can deduce that if all horizontally moving objects they have seen are “tuge” that this is a likely candidate for an unseen object moving horizontally as well. However, in the absence of further intervention, the languages which emerged were unable to differentiate between all scenes, and lacked expressivity since the words did not reflect an object’s shape or color. A second experiment then enforced the expressivity of the language by removing all but one of the scenes which were referenced by a common word between each generation. In this case, clear morphemes emerged which mapped onto distinct properties of the scenes. For example, all blue objects had the prefix “l-,” while bouncing objects had the suffix “-plo.” Thus, the generational transmission of language promoted regularity, but only with the enforced expressivity of the language did compositional morphemes (signals) emerge (9). Subsequent simulations and experiments have further highlighted the tension between compression and expressibility as a driving pressure behind the structure of natural language (5).

Due to the clear benefits to human cognition and language use, recent work in deep learning has aimed to understand whether artificial neural networks can efficiently generalize to novel stimuli by exploiting regularity in their datasets (17, 18). There has even been recent adoption of IL for machine learning tasks such as maintaining the structure of language when a pretrained model is used for a downstream task with Seeded IL (19), and Supervised Seeded IL (20). While deep learning techniques have made great strides in tasks like machine translation and language prediction, providing proof of principle that they can succeed in quasi-compositional domains (15, 21, 22), these methods are typically data hungry and often fail to generalize in simple settings when training data are scarce (1, 23). Similarly, while depth has been proposed as an architectural bias toward networks identifying compositional substructure (24), a number of studies have identified situations where depth alone is insufficient for structured generalization (18, 25–28). Another significant architectural factor is modularity, which can enable a system to generalize when modules are appropriately configured (29–33). However, identifying the right modularity through learning remains challenging (28, 34, 35) with IL being proposed as one algorithm which can learn to configure these modules (29). Thus, both empirical and theoretical work has shown that the generalization abilities of deep networks depends on a complex interplay of learning dynamics (36), architecture (23), initialization (37), and dataset structure (28).

In this work we use the deep linear network framework of Saxe et al. (36, 38) to analyze the ability of IL to produce compositional language with artificial neural networks. Our results provide insight into the ability of ML models to become systematic, confirm the necessity of multiple generations of IL for deep linear networks, and demonstrate that depth in the networks provides an inductive bias that enables IL to identify compositional structure in data. We begin by describing the general IL dynamics in shallow networks and compare these results to a prior Bayesian model using Markov chains (4, 14). Motivated by the success of deep linear networks in modeling child semantic learning (38) we then consider the impact of depth on the IL process which provides the primary conceptual and technical contribution of this work. We then follow this with an analysis of systematicity in the deep linear networks which arises from IL.

## Iterated Learning Dynamics of Shallow Neural Networks

The first step to establish whether generations of gradient descent learners have an implicit bias toward systematicity is to obtain closed-form learning dynamics for neural networks in the IL procedure. We build on known exact solutions to the dynamics of learning from small random weights in linear networks (36, 38) to describe the full learning trajectory analytically. Conceptually, our aim is to understand the input–output mapping of the network throughout training. When the input to the network represents a stimulus or meaning to be expressed, and the output is the description or signal to express that meaning, then this mapping from meaning to signal is the network’s language. By writing these dynamics in terms of the singular value decomposition below, we are able to connect this language and its evolution over training and generations to distinct “concepts” or patterns in the data (reflected by the singular vectors). By analyzing how learning over generations favors certain patterns, we can begin to understand how the inductive biases of learners will influence the evolution of language. If the network favors meaning–signal mappings which allow the meaning to be described by its components, then the network favors compositional structure in the data (2) and we consider this systematicity by the linear network (28, 39).

Consider a shallow neural network, with weight matrix W computing output ${\hat{Y}}_{i}=WX_{i}$ where ${\hat{Y}}_{i}\in R^{D}$ (the produced signal), in response to an input data point $X_{i}\in R^{N}$ (the meaning to be expressed) from a dataset (X,Y) (where $Y_{i}$ is the “correct” signal the network is learning to produce) with P data points, and trained to minimize the quadratic loss using full batch gradient descent $L\left(W\right)=\sum_{i=1}^{P}\frac{1}{2}\left|\right|Y_{i}-WX_{i}{\left|\right|}_{2}^{2}$. We can derive the learning dynamics of W and show that it depends on the singular value decomposition (SVD) of the correlation matrices: $\Sigma^{x}=\frac{1}{P}XX^{T}$ and $\Sigma^{\mathrm{yx}}=\frac{1}{P}YX^{T}$. Let $v^{\alpha }$ denote the α-th singular vector of the square $\Sigma^{x}$ matrix, and $u^{\alpha }$ denote the α-th left singular vector of $\Sigma^{\mathrm{yx}}$ with corresponding right singular vector $v^{\alpha }$. Similarly, $\delta_{\alpha }$ denotes a singular value of $\Sigma^{x}$ and $\lambda_{\alpha }$ denotes a singular value of $\Sigma^{\mathrm{yx}}$. With reasonable assumptions (see *SI Appendix* for a full derivation), the learning dynamics can be described explicitly in terms of the dataset correlation matrices’ singular vectors:

[1] $$\begin{array}{c} W\left(t\right)=\sum_{\alpha =1}^{rk(\Sigma^{\mathrm{yx}})}\pi_{\alpha }\left(t\right)u^{\alpha }v^{\alpha T}. \end{array}$$

Here $\pi_{\alpha }\left(t\right)$ is the α-th effective singular value of the network’s mapping after t epochs of training, and $rk(\Sigma^{\mathrm{yx}})$ denotes the rank of the input–output correlation matrix. Importantly, the only terms in Eq. **1** which are time dependent are the individual scalar singular values ($\pi_{\alpha }\left(t\right)$). The trajectory of each effective singular value is described as

[2] $$\begin{array}{c} \begin{array}{cc} \pi_{\alpha }\left(t\right) & =\lambda_{\alpha }/\delta_{\alpha }\left(1-\exp\left(-\delta_{\alpha }t/\tau \right)\right)+\pi_{0}\exp\left(-\delta_{\alpha }t/\tau \right) \end{array} \end{array}$$

which begins at the initial value $\pi_{0}$ when t=0 and increases to $\lambda_{\alpha }/\delta_{\alpha }$ as t→∞. Importantly, the time course of learning depends on the singular values of the input correlation matrix, $\delta_{\alpha }$ as this is the coefficient of time (t) in the equation (38). Since we begin training with small weights, $\pi_{0}$ will always be less than the asymptotic value ($\pi_{\alpha }$) until they converge. We define each combination of one singular value and its corresponding left and right singular vectors as a “mode”. Each mode defines an input–output mapping (language) in its own right and by adding multiple modes to this mapping a language will become increasingly complex. Since singular vectors can be considered concepts in the data, by learning a mode a learner is acquiring a portion of the desired language. Our analysis in this work relies on the relative speed at which modes start and finish learning. Since the singular value is the only portion of a mode that changes over time, “learning the singular value” corresponds to “learning the mode” and we can use these phrases interchangeably. It is helpful to introduce two terms: First, “escaping time” (denoted by ${\hat{t}}_{\alpha }$) is the time taken for a mode to begin learning (grow meaningfully larger than 0): $\pi_{\alpha }>\rho$ for a small value of ρ∈R. Second, “hitting time” (denoted by $t_{\alpha }^{*}$) is the time taken for a mode to converge to its final value: $\pi_{\alpha }-\left(\lambda_{\alpha }/\delta_{\alpha }\right)<\rho$. We derive explicit equations for ${\hat{t}}_{\alpha }$ and $t_{\alpha }^{*}$ in terms of the dataset singular values in *SI Appendix*.

Saxe et al. (38) use Eq. **1** to study semantic learning in children and show that for a structured dataset each of the terms defining the network training dynamics take on an interpretable meaning. For example, when given a dataset of 4 items and tasked with predicting the properties of a given item [an abstraction of a common type of cognitive test (40)], the network will learn to identify the underlying structure of the data defined by the properties. The canonical example forms a hierarchy by having a common property of “grow” which is active for all items, while half the items can “move” and the other half have “roots” forming the distinction between animals and plants. Each item is then given a unique feature forming the bottom level of the hierarchy. In this case, the right singular vectors ($v^{\alpha T}$) align to identify different levels of the hierarchy, while the left singular vectors ($u^{\alpha }$) align to the corresponding set of typical output properties for each level of the hierarchy. Finally, the singular value from the dataset ($\lambda_{\alpha }$) is the strength of the association between these input and output “concepts,” and the network singular value ($\pi_{\alpha }$) reflects its learned association between the concepts. For our purposes, the learning of these concepts corresponds to the learning of patterns or structure in the meaning–signal mapping of a language.

**Fig. 1.**
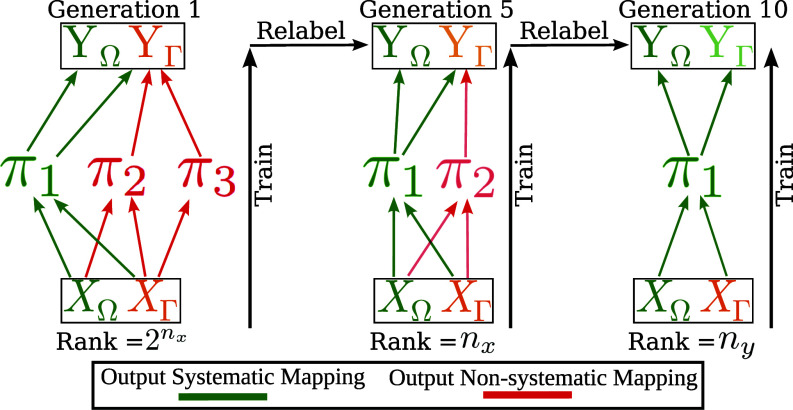
The iterated learning procedure: Generations of agents learn to map meanings (X) to signals (Y) generated by their parent, and pass on the meaning to signal examples to their children. The portions of the mapping (language) which are easier to learn are maintained over generations, while difficult language is lost. We demonstrate this process on a linear neural network and prove that IL is able to refine the language to depend on a minimal set of necessary concepts (the $\pi_{1}$ modes) to incur no error on the compositional output component of the signals ($Y_{\Omega }$). By reducing the rank of the mapping (removing unnecessary modes) while maintaining the compositional portion of the signals ($Y_{\Omega }$) the final language balances regularity and expressivity. By exploiting the compositional structure in the meanings ($X_{\Omega }$) and signals ($Y_{\Omega }$) the network is displaying systematicity. This figure also summarizes the notation, structure, and singular value decomposition of our space of datasets which is key for the theoretical results which follow. $n_{x}$ and $n_{y}$ are the number of compositional components of the meaning and signal spaces respectively, while $X_{\Gamma }$ and $Y_{\Gamma }$ are the noncompositional portions of the meaning and signal spaces respectively. Importantly, the lowest the network rank can go without losing unnecessary expressivity is $n_{y}$ although some expressivity will be lost by design if $n_{x}<n_{y}$. Settings where $n_{x}=n_{y}$ will not lose expressivity.

With IL each generation learns from the “language” acquired by the previous generation (see Fig. 1 for a depiction of the IL procedure). To instantiate this setting with a learning bottleneck so that a learner is unable to memorize the full initial language within a single generation, we start from a particular dataset, but halt training before full convergence after a predefined number of training steps. We then use the network’s outputs (logits) as the target outputs for the next generation. From very early on in training, learning occurs along the modes of variation (the concepts) determined by the singular vectors in Eq. **1** and the dataset’s singular vectors will be maintained for all generations. Thus, for all generations, the network’s input–output mapping (language) takes the same form as the original dataset’s singular value decomposition, just with changing singular values (associations between concepts). What this structure looks like depends on the exact instantiation of the initial dataset which we leave general for now. Noting this fact permits an analysis of iterated learning dynamics by tracking the change in the dataset singular values, corresponding to the amount of learning progress made by the previous generation’s agent. Thus, for generation G>0 of learning the asymptote of the network’s mapping ($\lambda_{\alpha }^{G}/\delta_{\alpha }$) is equal to the effective singular value of the network at the end of the previous generation of training ($\pi_{\alpha }^{G-1}$). Here $\lambda_{\alpha }^{0}$ and $\delta_{\alpha }^{0}$ are the singular values from the original dataset. Thus, by a recursive application of Eq. **2** we can model the full dynamics of iterated learning:

[3] $$\begin{array}{c} \begin{array}{cc} \pi_{\alpha }^{G}\left(t\right) & =\lambda_{\alpha }^{G}/\delta_{\alpha }\left(1-\exp\left(-\delta_{\alpha }t/\tau \right)\right)+\pi_{0}\exp\left(-\delta_{\alpha }t/\tau \right)\\ & =\pi_{\alpha }^{G-1}\left(1-\exp\left(-\delta_{\alpha }t/\tau \right)\right)+\pi_{0}\exp\left(-\delta_{\alpha }t/\tau \right). \end{array} \end{array}$$

From Eq. **3** we conclude that a shallow network is not a sufficient model for IL, as the modes show an exponential approach to their asymptote and all modes are learned at once. Consequently, from the first generation of IL information on all modes will be lost. This corresponds to portions of all structure in the language being forgotten during each generation and over enough generations the initial language will be forgotten altogether. This also reiterates prior conclusions from Bayesian models of IL using Markov chains which found that over sufficiently many generations the language collapsed to be completely determined by the prior distribution over possible languages (4, 14). We briefly review these models and compare them to our model now.

## Comparison to Prior Models of IL

The prior Bayesian models (4, 14) defined a hypothesis space h, or the space of possible languages, and the data (d) which could be produced from such a language P(d|h). By placing a prior distribution over the hypothesis space P(h) it is possible to define a joint distribution which describes the transmission of language over one generation as P(h,d)=P(d|h)P(h). This distribution reflects the change in belief about a language which occurs when a learner is only exposed to the language by a sampling of data from it. By marginalizing over the possible datasets which could be produced, they obtain a distribution over languages forming a Markov chain where the change in the language over multiple generations is $\sum_{d}P\left(h_{g+1}|d\right)P\left(d|h_{g}\right)$ and g denotes the generation. While Griffiths et al. (14) consider multiple sampling strategies from the posterior distribution, the most immediate comparison to our shallow neural network is using maximum a posteriori (MAP) estimation which chooses the language with the highest posterior probability. In Griffiths et al. (14) and Kirby et al. (4) it was found that over generations the impact of the initial dataset was lost and the distribution over languages would be defined purely by the prior probability.

To illustrate our model, we can draw comparisons with these Bayesian models (4, 14). First, our corresponding component to their data variable (d) is the dataset which we use to train our model (X and Y). In the Bayesian models, the language (h) is encoded as a set of variables and the distribution over the candidate languages is determined explicitly from the data (d). In our model the language is the input–output mapping of our neural network (W) and the distribution over languages is the distribution over the parameters of the network which form this input–output mapping. Crucially, and unlike the Bayesian model, to obtain the language (network parameters W) from data we do not infer these parameters directly from the data but rather use a training algorithm: full batch gradient descent to minimize the quadratic loss. We do not include an explicit prior on our model parameters, and so we do not have a term corresponding directly to the Bayesian model’s prior over the hypothesis space (P(h)). However, the small initialization of our network parameters plays a similar role to a Gaussian prior centered at 0. Thus, the fact that our model forgets the correct signals for the dataset entirely over the generations, and the distribution over weights collapses to the small initial weight distribution, is in agreement with the prior models. Beyond allowing a more literal interpretation of language being the mapping from meaning to signal, the use of a learning algorithm to determine the network weights (the language) from data allows us to understand how learning dynamics affect the IL framework. Specifically, by not allowing the learning algorithm sufficient time to memorize the input–output mappings in the dataset we can determine the effect of the learning bottleneck on IL. Second, as we are using a model of learning which has previously been used to understand semantic learning in children (38), we can draw comparisons to how the known properties of child semantic learning (specifically the learning dynamics) influence IL.

Our model does have some limitations compared to the Bayesian models (4, 14) which are needed to ensure that we can obtain an equation for the dynamics of IL, such as Eq. **3**. First, our model does not sample a subset of the data when training and it relabels the entire dataset for a subsequent learner. Thus, our network does not model the transmission bottleneck of IL which also serves an important pressure on a language to be easily learnable (41, 42). Conceptually, our model is in a setting where it is possible for a learner to experience all meaning-to-signal pairs (all data points in the dataset) but is not given sufficient time to memorize all such pairs. Second, our model is not sampling discrete values for the signals (sampling P(d) in the Bayesian model) of each data example but rather learning continuous values for the signals (Y) associated with each meaning (X). This is a necessary simplification to obtain the learning dynamics equations as discretizing the data by sampling can change the structure of the singular value decomposition of the dataset. As we will show in the following section, the compositional portion of a dataset tends to be significantly lower-rank than the rest of the dataset, meaning that significantly fewer data points need to be seen for this portion of the dataset structure to remain across generations. This means that the compositional portion would not change even if we were discretizing the signals and the conclusions we draw in subsequent sections are likely to generalize, especially as the size of the dataset grows. Finally, our model is not sampling from a space of languages (sampling P(h) in the Bayesian model) and it has no mechanism to introduce new signals (morphemes or words) into the language. Thus, structured portions of the language must be present from the first generation. While human creativity certainly plays a role in language evolution (9), we are focused here on the effect of learning dynamics and a learning bottleneck on IL. Thus, we do not consider how structure appears in a language, rather we are concerned with a learner’s ability to identify and exploit this structure when it is there to aid learning. Overall, our model provides a complementary set of benefits and limitations to the Bayesian models (4, 14) by focusing on the learning bottleneck of IL rather than the transmission bottleneck (41, 42). Yet we can find commonalities between the two types of models and reproduce the collapse toward a degenerate language from the Bayesian models in our shallow neural network. An impactful direction for future work would unify the transmission and learning bottlenecks into a single model more formally.

Having described our setup with a shallow neural network and reviewed the prior Bayesian models of IL (4, 14), we now turn our attention to the addition of depth into the neural network architecture. This step is motivated by the findings of Saxe et al. (38) which showed that architectural depth is key to reproduce some of the established behavior of semantic learning in children. Thus, we hypothesize that these more realistic learning dynamics of semantic cognition will provide helpful inductive biases that also translate into a more faithful model of language acquisition and cultural transmission.

## Iterated Learning Dynamics of Deep Linear Networks

While deep linear networks can only represent linear input–output mappings, the dynamics of learning change dramatically with the introduction of one or more hidden layers (36, 38, 43, 44, 45), and the learning problem becomes nonconvex (46). They therefore serve as a tractable model of the influence of depth specifically on learning dynamics, which prior work has shown to impart a low-rank inductive bias on the linear mapping (47). Following a similar process to the analysis of shallow neural networks, the mapping by a deep network can be written as

[4] $$\begin{array}{c} W^{2}\left(t\right)W^{1}\left(t\right)=\sum_{\alpha =1}^{rk(\Sigma^{\mathrm{yx}})}\pi_{\alpha }\left(t\right)u^{\alpha }v^{\alpha T}, \end{array}$$

where $W^{1}$ and $W^{2}$ are the input-to-hidden and hidden-to-output layers of the neural network respectively. The only difference between the deep and shallow networks is the dynamics of the network effective singular values. The generational dynamics of an effective singular value in the deep linear network is described as

[5] $$\begin{array}{cc} \pi_{\alpha }^{G}\left(t\right) & =\frac{\lambda_{\alpha }^{G}/\delta_{\alpha }}{1-\left(1-\frac{\lambda_{\alpha }^{G}}{\delta_{\alpha }\pi_{0}}\right)\exp\left(\frac{-2\lambda_{\alpha }^{G}}{\tau }t\right)}\\ & =\frac{\pi_{\alpha }^{G-1}}{1-\left(1-\frac{\pi_{\alpha }^{G-1}}{\pi_{0}}\right)\exp\left(\frac{-2\lambda_{\alpha }^{G}}{\tau }t\right)}. \end{array}$$

Once again the network is trained from small initial weights. It is key to note that the time-course of the trajectory is now dependent on the $\Sigma^{\mathrm{yx}}$ singular values ($\lambda_{\alpha }$). Thus, unlike the shallow network, $\Sigma^{x}$ affects the stable point of the network singular values but not the rate of learning. We also assume that $n_{h}>rk\left(\Sigma^{\mathrm{yx}}\right)$ where $n_{h}$ is the number of hidden neurons. If this is not the case, then the model will only learn the top $n_{h}$ singular values of the input–output mapping (36).

Deep linear neural networks display three common traits typical of semantic learning in children: 1) stage-like transitions, 2) progressive differentiation and 3) illusory correlations (48). This is in contrast to shallow neural networks which do not display any of these three properties (38). Stage-like transitions refers to the trend where children learning new concepts are initially slow to acquire the knowledge but reach an insight-like moment where it is suddenly learned (49) and integrated into their understanding of the world (50). Progressive differentiation refers to the trend of children developing skills sequentially and in a hierarchical order where earlier skills support the learning of subsequent ones (51, 52). Finally, illusory correlations describe how children tend to overgeneralize new concepts when they are recently acquired. Importantly, this behavior has been observed in children learning language (53). For example, a child who has recently learned the suffix “-est” from the word “nicest” will incorrectly extrapolate this to the word “good” and say “goodest.” Moreover, it was shown that the probability of overgeneralization occurring in children is linked to the similarity of the new object to previously seen objects being acted on (53).

**Fig. 2.**
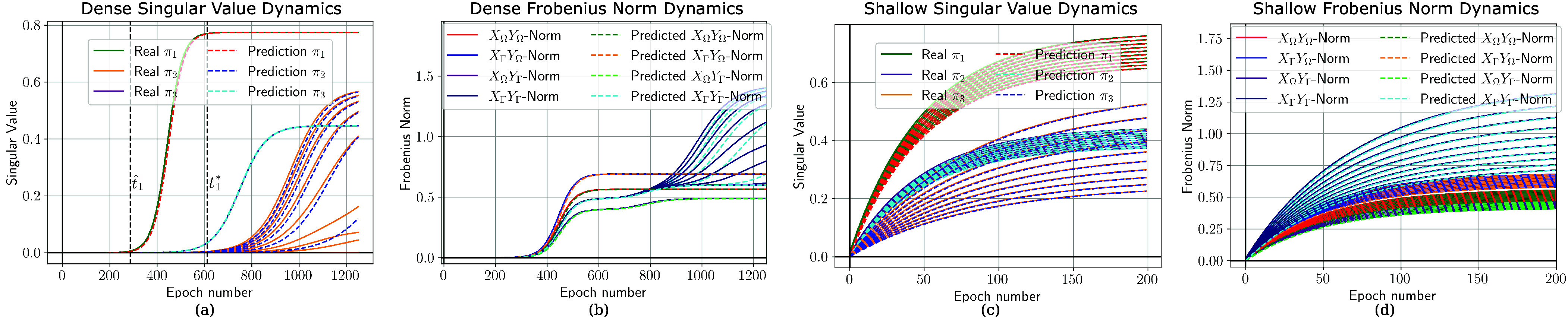
Analytical learning dynamics for deep (*A* and *B*) and shallow (*C* and *D*) linear networks. (*A* and *C*) Comparison of predicted and actual singular value trajectories over learning, for the three unique dataset singular values. ${\hat{t}}_{1}$ and $t_{1}^{*}$ denote the escaping time and hitting time respectively for the first mode of variation with ρ=0.005. (*B* and *D*) Comparison of predicted and actual Frobenius norms of the input–output mapping to/from compositional ($X_{\Omega },Y_{\Omega }$) and noncompositional ($X_{\Gamma },Y_{\Gamma }$) features. Deep networks show distinct stages of improvement over learning. However, at no point is a mapping learned which relies exclusively on compositional features or language. However, this setting depicts the progressive removal of the $\pi_{3}$ mode of variation over 10 generations. By the final generation of the dense network training the nonsystematic norms exhibit two stage-like transitions corresponding to the learning of the two remaining modes of variation. The shallow network does not learn the modes in separate stages and so the removal of one distinct mode is impossible without simultaneously removing portions of all other modes. This demonstrates the theoretical observations from the dynamics of IL. *Dataset Parameters*: $n_{x}=3,n_{y}=2,k_{x}=3,k_{y}=1,$ and r=2.

Each of the three properties of child semantic learning (48) can be linked directly to a behavior in deep linear networks through Eq. **5** and can be observed in the learning dynamics shown in Fig. 2*A*. Stage-like transitions occur due to the final functional form of the learning dynamics which define a sigmoidal trajectory. This sigmoidal shape reproduces the slow initial learning as the mode strength stays near its small initial value until reaching the sharp exponential increase before settling to its final value. Progressive differentiation occurs due to the learning speed of a mode depending on the input–output singular value of that mode ($\lambda_{\alpha }$). Thus, modes will be learned in order of the magnitude of their respective singular values. Finally illusory correlations occur as a result of progressive differentiation, where learned information from the faster modes is overgeneralized until the subsequent and more specific modes are learned to correct the residual error. For a given dataset, each of these behaviors can take on clear interpretations. In the hierarchical dataset progressive differentiation means that distinctions in the hierarchy are learned in order, where the difference between the properties of plants and animals are learned before the differences between types of plants and types of animals. This leads to illusory correlations where the property of one plant is attributed to all of them before the network has learned to distinguish types of plants.

### The Requirement of Depth.

Having described our framework for modeling IL and reviewed the connection of deep linear networks to child semantic learning, we can now demonstrate our first key finding: that architectural depth is necessary for the IL process to refine a language toward regular structure without losing expressivity, in linear networks. Within our framework we are able to define a number of key concepts in the study of IL (8, 54). The pressure of IL to refine a language toward regularity corresponds to its ability to remove modes (concepts) from the dataset by keeping a mode’s singular value near 0. Thus, the rank (number of modes/concepts) of the input–output mapping (language) which needs to be learned by the network decreases over generations, making it easier to learn. However, as the rank drops, so too does the number of unique labels which can be given to items in the dataset. Thus, expressivity can be measured by the number of unique labels (signals) and can decrease over generations of IL. This makes explicit the effect seen by Kirby et al. (9) where IL is able to produce regular languages at the expense of expressivity in the absence of additional pressures. To maintain expressivity, we define “maintained modes,” which are modes of variation (and the corresponding concepts) that our model should not change over generations. We can maintain expressivity then by ensuring that the network mapping is always of sufficient rank to give a unique label (signal) to each item. To measure this trade-off between regularity and expressivity for a dataset used to train a linear neural network we introduce the Rank-Cardinality Ratio in *Definition* 1:

Definition 1: **Rank-Cardinality Ratio (RCR)**:

$RCR\left(X,Y\right)=\frac{rk(YX^{T})}{\min\{|X|,|Y|\}}\approx \frac{\text{regularity}}{\text{expressivity}}$

The RCR is helpful as it provides a metric of how easy it is to generalize on an arbitrary linearly separable dataset. The rank of the correlation matrix $rk(YX^{T})$ determines how many noncolinear data points are needed to learn the correlation matrix. The minimum of the input and output cardinality determines how many unique data points are in the dataset. Thus, the maximum RCR of 1 means all data points must be seen to learn the correlation. The closer the RCR gets to 0 the relatively fewer training examples are needed to generalize to a larger dataset. Thus, this metric summarizes the ratio between how many data points must be seen for generalization compared to how many data points are learned in total. Following the findings of Kirby et al. (9), we should see compositionality emerge in a language when the network is made to balance regularity and expressivity. This is the primary consideration of the next section once we have defined a dataset.

The necessity of depth for the IL procedure follows directly from the stage-like transitions property of semantic learning. Stage-like transitions in the deep linear networks allow for one mode (concept) to be learned while another develops no association (the mode’s singular value remains near 0). Consequently, it is possible to find a time where a faster mode has converged while another is still learning. Over sufficient generations the converged (maintained) mode will remain unchanged while the slower mode is forgotten. By introducing a learning bottleneck (ending a generation of training before the convergence of some effective singular values) we also decrease the input–output singular values for the next generation. Since these singular values also determine how quickly the mode is learned, this means the mode will be learned slower for subsequent generations. Consequently, the rate that a mode is forgotten increases with every generation, further supporting IL. Progressive differentiation means that the more specific modes with a smaller singular value will be forgotten and result in illusory correlations being introduced into the labels. These illusory correlations provide the mechanism to change the language over generations and promote regularity. In contrast, shallow network modes show an exponential approach to their asymptote and all modes are learned at once. Thus, there will never be an opportunity for IL to remove a mode without also losing information on modes which we aim to maintain. Consequently, IL in the shallow networks can only lead to an overly regular language, and eventually no language at all. Overall, the stage-like transitions mean that, unlike shallow models (14), deep linear networks can find a stable language which is still expressive.

We also find that there is an optimal depth for IL in Informal *Theorem* 1 (formal theorems are left to *SI Appendix*). This occurs when the modes learn the slowest as this makes the modes as separable as possible due to the sigmoidal shape of the learning dynamics. The benefit is that IL will be able to more quickly remove the unwanted modes of variation as these modes will be closer to their initial value when the maintained modes have converged. We find that for a reasonable initial mode strength of $\pi_{0}=0.001$ the optimal depth is D≈15 layers.

Theorem 1. *Given a dataset* (X,Y) *in the deep linear network setting, having a depth of* $D\approx 1-2\ln(\pi_{0})$ *maximizes the distance between the escaping time of the modes, making IL as efficient as possible.*

**Proof Sketch**: To prove this result, we derive the general dynamics for deep linear networks with depth now greater than two layers (D≥2). This results in a dynamics reduction which includes depth as a term that impacts the speed that the modes learn. From this we find the critical point in terms of D and show that learning speed at initialization is minimized when $D=1-2\ln(\pi_{0})$.

### The Requirement of Multiple Generations.

A lingering question in the use of IL for machine learning has been whether having multiple generations of learners is actually necessary. This is in contrast to a hypothetical optimal early stopping point which would provide all the same benefits as IL but within a single generation (11, 55). *Theorem* 2 answers this question for a deep linear neural network:

Theorem 2. *Given a dataset* (X,Y) *in the deep linear network setting having multiple generations of learners is a necessary condition for guaranteed removal of only the desired modes of variation.*

**Proof Sketch**: To prove that an optimal single-generation early stopping point exists, we are required to show that the removable modes will not start learning (reach their escaping time) before the maintained modes have converged (reached their hitting time). In other words, we need to show that enough of the faster learning concepts required to maintain expressivity will be learned completely before the concepts we aim to remove can begin to be learned. We show that this is not true in general for one generation (G=0) using a contradicting example. Second, we show that this will be true after a sufficient number of generations G. The key step toward this is showing that the time taken for a removable mode to converge is larger than the time taken by the maintained mode to converge—a significantly easier condition than comparing convergence time of maintained modes with the time taken to start learning by the removable modes. Noting the difference in convergence times is enough for IL to be applicable. Once IL is applicable then as G→∞ the removable mode will learn slower over the generations due to the successive decrease in its singular value and the escaping time becomes larger than the maintained mode hitting time (which has a consistent singular value), proving the theorem.

## Formalizing Systematicity for Deep Linear Networks

Having established the training dynamics for IL and the necessity for both depth and multiple generations for IL to be effective, we now turn our attention to establishing the benefit of IL for systematicity and in producing compositional language. To do so we must formalize a space of datasets which display the inductive biases of learning. We build on prior work which provides such a space of datasets (28).

### A Space of Analyzable Datasets.

The space of datasets was designed to capture key aspects of common benchmarks for assessing systematicity of large nonlinear (often modular) artificial neural networks, such as SCAN (56) and gSCAN (39), among others (57, 58). To be more applicable to the linguistic background of IL, we rephrase the space of datasets in terms of the mapping from logical forms (our internal, potentially semantic, representation of the world) to orthographic forms (words or sentences) commonly discussed in linguistics (12). For brevity, we have been calling the logical form “meaning” and the orthographic form “signal.” The fundamental aspect of the analysis, however, remains the same: We use a space of datasets parameterized by the degree of compositional and noncompositional structure. We then use the closed-form SVD for all datasets in the space (written in terms of the dataset parameters) to establish how dataset structure affects the inductive bias of the neural network learning dynamics.

**Fig. 3.**
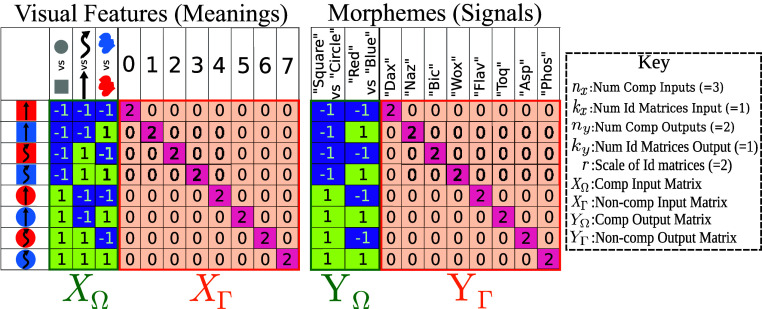
We schematize the setting with a space of datasets containing compositional ($X_{\Omega }$) and noncompositional ($X_{\Gamma }$) components in the input meanings (*Left* panel). The network’s task is to map from a meaning to a signal for each object, forming a language. The meaning–signal mapping (language) could be learned by the composition of descriptive words ($Y_{\Omega }$) for example “the small red square” or by memorizing a name for each object ($Y_{\Gamma }$), for example “the bic.” *Dataset Parameters*: $n_{x}=3,n_{y}=2,k_{x}=1,k_{y}=1,$ and r=2.

The space of datasets can be compared to the “alien” object-description task of Kirby et al. (9) and the task must remain linearly solvable to allow for theoretical analysis. An example of one dataset from the space of datasets is depicted in Fig. 3. The compositional visual features (which give meaning) in this example are the shape, color, and movement of the object. The noncompositional visual feature is a unique digit associated with the image and affords the network the ability to consider the object as a whole. This is then mapped to the signal, which expresses meaning, with compositional portions of the signal corresponding to each object feature. Additionally, the object is allocated a unique name as the noncompositional portion of the signal and the network has the choice of how it uses the compositional and noncompositional portions of the signal to describe the objects. Finally, in our space of datasets we account for the fact that not all visual features need to be used for naming. For example, whether a chair is made of plastic, wood, or metal is not relevant to calling it a chair. Thus, the set of signal features may be smaller than the set of visual or semantic features.

To formalize this setting, Jarvis et al. (28) define a parametric space of datasets with input and output matrices $X={\left[X_{\Omega }\;X_{\Gamma }\right]}^{T}$ and $Y={\left[Y_{\Omega }\;Y_{\Gamma }\right]}^{T}$ respectively, where $n_{x},n_{y},k_{x},k_{y},r\in Z^{+}$ are the parameters that define a specific dataset. The compositional input feature matrix $X_{\Omega }\in {\left\{-1,1\right\}}^{2^{n_{x}}\times n_{x}}$ consists of all binary patterns with $n_{x}$ bits. This means that the dataset contains $2^{n_{x}}$ examples. The compositional output feature matrix $Y_{\Omega }\in {\left\{-1,1\right\}}^{2^{n_{x}}\times n_{y}}$ is a subset of $X_{\Omega }$ with $n_{y}$ features (columns). Next, the noncompositional input feature matrix $X_{\Gamma }=\left[rI_{1}\;...\;rI_{k_{x}}\right]$ consists of $k_{x}$ scaled identity matrices, $I_{i}\in {\left\{0,1\right\}}^{2^{n_{x}}\times 2^{n_{x}}}$. Similarly, the noncompositional output matrix $Y_{\Gamma }=\left[rI_{1}\;...\;rI_{k_{y}}\right]$ has $k_{y}$ scaled identity matrices $I_{i}\in {\left\{0,1\right\}}^{2^{n_{x}}\times 2^{n_{x}}}$ with scale factor r. These identity matrices provide a single feature for each pattern which is only on for that pattern. Crucially for our analysis, the amount of compositional structure can be titrated by adjusting $n_{x}$ and $n_{y}$. Similarly, $k_{x},k_{y}$ and r control the frequency and intensity of the noncompositional features, which are both factors that can promote noncompositional language being used by humans (8, 48).

As described above, the network’s total input–output mapping at all times in training is a function of the singular value decomposition of the dataset statistics. For all datasets in the space there are three distinct input–output correlation ($\Sigma^{\mathrm{yx}}$) singular values $\lambda_{1}$, $\lambda_{2}$ and $\lambda_{3}$ and therefore three asymptotes $\pi_{1},\pi_{2}$ and $\pi_{3}$, shown in Eqs. **8** to **13**. Similarly, the input correlation ($\Sigma_{x}$) singular values are shown in Eqs. **6** and **7**. Note that the singular values are written in terms of the five dataset parameters, which allows for an analysis of how dataset structure influences the network training dynamics (28) and inductive bias of IL. Substituting these expressions into the dynamics equations (Eq. **3** for the shallow network and Eq. **5** for the deep network) and by writing this decomposition in terms of the dataset parameters we obtain equations for the networks mapping and full learning trajectories across all generations, at all times in training and for all datasets in the space.

[6] $$\begin{array}{c} \delta_{1}=\frac{\left(k_{x}r^{2}+2^{n_{x}}\right)}{2^{n_{x}}} \end{array}$$

[7] $$\begin{array}{c} \delta_{2}=\frac{k_{x}r^{2}}{2^{n_{x}}}. \end{array}$$

[8] $$\begin{array}{c} \lambda_{1}={\left(\frac{\left(k_{x}r^{2}+2^{n_{x}}\right)\left(k_{y}r^{2}+2^{n_{x}}\right)}{2^{2n_{x}}}\right)}^{\frac{1}{2}} \end{array}$$

[9] $$\begin{array}{c} \pi_{1}=\lambda_{1}/\delta_{1}={\left(\frac{k_{y}r^{2}+2^{n_{x}}}{k_{x}r^{2}+2^{n_{x}}}\right)}^{\frac{1}{2}} \end{array}$$

[10] $$\begin{array}{c} \lambda_{2}={\left(\frac{\left(k_{x}r^{2}+2^{n_{x}}\right)\left(k_{y}r^{2}\right)}{2^{2n_{x}}}\right)}^{\frac{1}{2}} \end{array}$$

[11] $$\begin{array}{c} \pi_{2}=\lambda_{2}/\delta_{1}={\left(\frac{k_{y}r^{2}}{k_{x}r^{2}+2^{n_{x}}}\right)}^{\frac{1}{2}} \end{array}$$

[12] $$\begin{array}{c} \lambda_{3}={\left(\frac{k_{x}k_{y}r^{4}}{2^{2n_{x}}}\right)}^{\frac{1}{2}} \end{array}$$

[13] $$\begin{array}{c} \pi_{3}=\lambda_{3}/\delta_{2}={\left(\frac{k_{y}}{k_{x}}\right)}^{\frac{1}{2}} \end{array}$$

To empirically verify our theoretical results up to this point we simulate the full training dynamics for deep and shallow linear networks trained using gradient descent on an instantiation from the space of datasets with parameters $n_{x}=\;3,n_{y}=\;2,k_{x}=\;3,k_{y}=\;1,r=\;2$ (shown in Fig. 2). While training, we compute the singular values of the network after each epoch. These simulations of the training dynamics for each unique singular value are then compared to the predicted dynamics. We see close agreement between the predicted and simulated trajectories.^*^ Note the requirement of depth and multiple generations of IL to effectively remove a mode of variation without also losing information on other modes. The difference between deep and shallow network training dynamics can be seen by comparing the shape of the learning trajectories between Fig. 2 *A* and *C*. Note how $\pi_{3}$ is removed with the deep network (Fig. 2*A*) while $\pi_{1}$ and $\pi_{2}$ remain unchanged. In contrast, all modes are decreased with the shallow network (Fig. 2*C*) but none are removed.

An important property of the theoretical framework is that datasets in this space allow redundant solutions: The compositional output component can be generated based on compositional input features alone (systematic mappings), but they can equally be generated using noncompositional features alone, or some mixture of the two (nonsystematic mappings). This is reflective of a general fact: that in many settings, there are multiple ways to solve a problem (61). However, it is not the case that all approaches generalize equally well. This means that the inductive biases placed upon a model which influence the kinds of mappings it learns are a key consideration, even if the difference is not apparent on training data (14, 61).

The second important point to this framework is that different substructures in the data have different ranks. For example, the correlation between the compositional input and output features is a rank $n_{x}$ matrix. Thus, even though there are $2^{n_{x}}$ data points, we would only need a training set with $n_{x}$ noncolinear data points to generalize to all $2^{n_{x}}$ data points [this occurs with high probability when random sampling and as $n_{x}$ gets larger (28)]. Conversely, the mapping between noncompositional input and output features will always have a rank $2^{n_{x}}$ correlation. Thus, any generalization is impossible on this substructure as we need to see all data points to learn the correct correlation. Taken together, these two properties of the space of datasets mean that which of the possible mappings (solutions or languages) the network favors while learning will have very different rank properties. As a result different languages will have very different generalization properties. A similar argument has been made by Brighton (42) which notes that while insufficient stimuli are available to children to learn arbitrary languages, sufficient stimuli may be available to learn compositional ones due to the ease of generalization such compositionality affords. The RCR formalizes this notion into a metric for the languages learned by a linear network.

### Expanding the Definition of Systematicity.

In machine learning, it has been challenging to formalize the intuitive notion of systematicity demonstrated on accepted evaluation methods (23, 54, 57, 62, 63), with all prior definitions being domain specific (to language for example) or remaining practically intract- able (28, 64, 65). In most cases neural networks do not manage to generalize systematically without the addition of modular architectures, explicit regularizers, or a degree of supervision of the learned features (34, 39, 66). This includes transformer architectures which require highly structured datasets or prompting (67–69) to display systematic behaviors consistently. There are even counterexamples which find only a weak correlation between compositionality and generalization (64) or learning speed (70), reflecting a long-standing theoretical debate stretching back to the first wave of connectionist deep networks (26, 71–76).

Noting the difference in generalizability from relying on different substructure in data, Jarvis et al. (28) define systematicity as the identification and exploitation of the low-rank substructure to support generalization. This definition relies on the model identifying substructure in the input and output simultaneously to achieve systematicity. IL presents an example of where this is needlessly strict as IL only operates on the output labels of the network. However, IL has no mechanism to deal with noncompositional input structure aside from the inductive bias of gradient descent, which has been shown to be ineffective (28). Thus, in a similar manner to the ordered definition of systematicity from a specifically linguistic context (76), we provide a mathematical taxonomy of systematicity in linear networks, to better characterize the benefits of IL for systematicity. Specifically, we define output and input systematicity (*Definition* 2 and 3 respectively) as two independent forms of systematicity. By being both input and output systematic an agent achieves full systematicity (*Definition* 4), the higher level of the taxonomy. All three definitions rely on the Rank-Cardinality Ratio (RCR) in *Definition* 1. By defining systematicity in terms of the RCR it makes explicit that a systematic mapping uses substructure in the dataset that displays more regularity for a given level of expressivity.

Definition 2: **Output systematic generalization**: the reliance on substructure $Y_{\theta }$ such that $RCR\left(X,Y_{\theta }\right)<RCR\left(X,Y\right)$.

Definition 3: **Input systematic generalization**: the reliance on substructure $X_{\theta }$ such that $RCR\left(X_{\theta },X_{\theta }\right)<RCR\left(X,X\right)$, when $rk\left(X_{\theta }X_{\theta }^{T}\right)\geq rk\left(YX_{\theta }^{T}\right)$.

Definition 4: **Full systematic generalization**: the reliance on substructure $X_{\theta }$ and $Y_{\theta }$ such that $RCR\left(X_{\theta },X_{\theta }\right)<RCR\left(X,X\right)$ and $RCR\left(X_{\theta },Y_{\theta }\right)<RCR\left(X,Y\right)$, when $rk\left(X_{\theta }X_{\theta }^{T}\right)\geq rk\left(Y_{\theta }X_{\theta }^{T}\right)$.

Output and full systematicity make no claims on how the remaining portion of the output space should be learned or handled. Since systematicity is the identification of and reliance on low-rank substructure to generalize when available, exceptionally high-rank portions of the dataset will always require many data points to learn. What matters here is that these high-rank complex portions do not inhibit generalization to the lower-rank substructure as they would by increasing the overall rank of the dataset. By these definitions, IL does not assist with input systematicity as it does not affect the input feature space, either in the time span of a forward pass (attention), over epochs (learning) or over generations. Consequently, IL also does not assist with full systematicity.

### The Benefit of Iterated Learning.

Having obtained the IL dynamics and refined the definition of systematicity, we ask what type of content is learned first and whether output systematic mappings emerge at any point during training. To answer the first question, we present *Observation* 5. This observation shows that for the entire space of datasets, predominantly compositional substructure is learned first. Specifically, the mode (concept) connecting compositional input and output substructure ($\pi_{1}$) is learned fastest. Conversely, the modes which do not connect compositional substructure ($\pi_{2}$ and $\pi_{3}$) are learned last. The consequence is that it is always possible to remove some noncompositional output substructure from the “language” while preserving all compositional substructure. See Fig. 1 for a visual description of this process. Thus, *Observation* 5 proves the benefit of IL for the entire space of datasets, and strongly supports the hypothesis that IL is the cause of the compositional nature of human natural language.

Observation 5. For all points in the space of datasets: $n_{x},n_{y},k_{x},k_{y},r\in Z^{+}$ the input–output correlation matrix $\Sigma^{\mathrm{yx}}$ singular values will be ordered as $\lambda_{1}>\lambda_{2}>\lambda_{3}$.

**Proof Sketch**: The proof of this observation uses Eqs. **8**, **10**, and **12** and shows that there is no configuration of dataset parameters such that Eq. **8** ($\lambda_{1}$) is not the largest value and Eq. **12** ($\lambda_{3}$) is not the smallest.

From *Observation* 5 we are able to determine that IL is output systematic in terms of *Definition* 2. We summarize this in Observation 6.

Observation 6. For all points in the space of datasets: $n_{x},n_{y},k_{x},k_{y},r\in Z^{+}$, IL identifies exploitable low-rank substructure in the input–output correlation ($\Sigma^{\mathrm{yx}}$) and is output systematic.

**Proof Sketch**: From *Observation* 5, we know that the maintained modes will start learning (reach their hitting time) before the removable modes from the first generation of learning. Thus, due to *Theorem* 2 we know there is some G>0 where it is possible to select a time $t^{*}$ such that $\pi_{2}^{G}\to 0$ and $\pi_{3}^{G}\to 0$ while $\pi_{1}^{G}=\pi_{1}^{0}$. Thus, IL is able to remove all of the removable modes. We then demonstrate that the mapping identified by IL fits the definition of output systematicity in *Definition* 2. We show that the rank of the new mapping without the removable modes has decreased but the new mapping is still able to produce a unique output for each input, maintaining the cardinality of the dataset. Thus, the RCR for the new mapping is lower than for a mapping learning the original dataset.

**Fig. 4.**
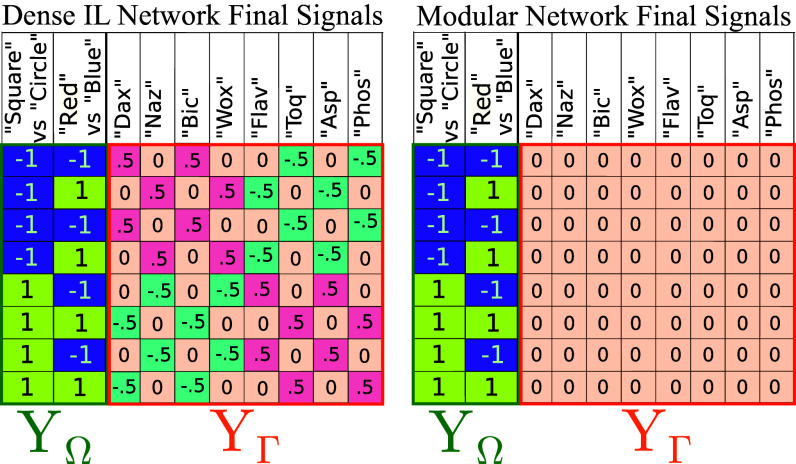
The final output signals from the IL algorithm (*Left*) for all meanings after removing modes $\pi_{2}$ and $\pi_{3}$ [comparable to the original output in Fig. 3 (*Right*)]. The IL network converges on a mapping (language) with rank $n_{y}=2$ (as depicted in Fig. 1) while maintaining some noncompositional components of the signals ($Y_{\Gamma }$). The modular network of Jarvis et al. (28) (*Right*) contains a module which only connects compositional meaning ($X_{\Omega }$) and signal components ($Y_{\Omega }$) and also has a rank of $n_{y}=2$. Thus, IL arrives at signals for each object with entangled compositional components (54) (replacing the noncompositional signal components ($Y_{\Omega }$) in Fig. 3 (*Right*) which was the identity block with highly structured values of 0.5 that correspond to different pairs of the compositional portion of the signals). This provides the same degree of regularity and expressivity as hand-crafted modularity.

We see that IL identifies an entirely different kind of substructure than that of Jarvis et al. (28). In Jarvis et al. (28) the identified substructure was between purely compositional components ($X_{\Omega }$ and $Y_{\Omega }$) and resulted from exclusively connecting these substructures of the input and output space with a neural module. The resultant input–output mapping between these substructures had a rank of $n_{y}$ even though there were $2^{n_{x}}$ data points and as a result it was possible to generalize on this substructure of the dataset. In the case of IL however, we can see from the singular vectors associated to $\pi_{1}$ that the full input and output remains connected (the full SVD for the space of datasets is shown in *SI Appendix* however Fig. 1 demonstrates the SVD structure). However, the input–output correlation once again converges on a rank of $n_{y}$. Thus, IL identifies a different form of substructure which has the same benefits for the regularity and expressivity of language as hand-crafted modularity. The final output of the IL algorithm using the dense network is compared to the compositional output from Jarvis et al. (28) in Fig. 4.

This echoes a similar point by Conklin et al. (54) which defines compositionality with variation: compositional substructure in a dataset which is obscured by a degree of natural language variation such as the use of synonyms. Conklin et al. (54) argues that commonly used measures of compositionality do not account for these variations even though the variation does not meaningfully change the ability of a network to generalize to unseen data. This leads to uncertainty on whether compositionality is correlated with superior generalization (64, 70). Transferring this to our framework would mean that the dataset shown after IL in Fig. 4 (*Left*) is a variation of the dataset which uses the compositional output features only, as it has the same rank (and hence regularity and generalizability) as these features. Specifically, the dataset after IL displays entanglement variability (54) as the remaining noncompositional features are changed to represent pairs of data points which entangles these features with the compositional ones. For ease, we will refer to this output structure as the entangled compositional output features. The fact that our measure of systematicity provides the same value to both strategies supports its use as a measure which does not suffer the same challenges with variability. However, the inability of IL to direct a model to ignore noncompositional input features is a limitation of the procedure which will hinder the model’s ability to generalize (28) even if the model is identifying highly regular and expressive (and therefore compositional) structure in the output (signals).

### Limitations of Iterated Learning.

While *Observation* 5 proves that IL is effective at identifying low-rank substructure in the output, IL has no mechanism to ignore noncompositional input features (see *SI Appendix* for a formal proof). This means that, if the network encounters an unseen data point, it would aim to rely on input features which are unique to other meanings to determine the appropriate signal. Consequently, the network will not produce the appropriate signal for the new meaning even if it is possible to determine the signal only from the compositional input structure. While our network is trained on the full dataset, we still aim for the network to use generalizable mappings (language). Thus, we now consider what is necessary to address this weakness of IL. Architectural modularity is one way of guaranteeing that noncompositional inputs cannot impact compositional output labels (28) [as shown in Fig. 4 (*Right*)]. However, this requires expert knowledge on how to define these modules and so we aim to find an alternate mechanism. To do so we consider the Frobenius norms of portions of the network mapping connecting the four main dataset substructures. The norms depicting the relation between the two input substructures with the output substructures (four in total) for multiple generations of learning are shown in Fig. 2. We are concerned with how much the noncompositional input features are used by the network to determine the compositional portion of the signal, as this is the portion of the language which does not generalize. The closed-form dynamics equation for the $X_{\Gamma }Y_{\Omega }^{G}$-Norm for all datasets in the space is shown in Eq. **14** and to remove the association between noncompositional input and compositional output we need $X_{\Gamma }Y_{\Omega }^{G}\text{-Norm}=0$. To do so we make *Observation* 7.

[14] $$\begin{array}{c} X_{\Gamma }Y_{\Omega }^{G}\text{-Norm}={\left(\frac{2^{n_{x}}n_{y}k_{x}r^{2}\pi_{1}^{2}\left(t\right)}{\left(k_{x}r^{2}+2^{n_{x}}\right)\left(k_{y}r^{2}+2^{n_{x}}\right)}\right)}^{\frac{1}{2}} \end{array}$$

Observation 7. For $n_{x},n_{y},k_{x},k_{y},r\in Z^{+}$, as $n_{x}\to \infty$ the association between noncompositional input and compositional output is removed: $X_{\Gamma }Y_{\Omega }^{G}\text{-Norm}\to 0$.

**Proof Sketch**: This can be shown by factoring out the terms of Eq. **14** which involve $n_{x}$. By doing this we see that the magnitude of this association is $O(\frac{1}{2^{n_{x}}})$ which decays to 0 as $n_{x}\to \infty$.

This observation demonstrates that, given an increasing number of input objects to be named, the network will favor compositional representations and avoid memorization. Thus, we provide one further extension of our taxonomy of systematicity by defining “weak full systematicity” in *Definition* 8. The definition can be extended similarly to input and output systematicity.

Definition 8: **Weak full systematic generalization**: the reliance on substructure $X_{\theta }$ and $Y_{\theta }$ such that $RCR\left(X_{\theta },X_{\theta }\right)<RCR\left(X,X\right)$ and $RCR\left(X_{\theta },Y_{\theta }\right)<RCR\left(X,Y\right)$, when $rk\left(X_{\theta }X_{\theta }^{T}\right)\geq rk\left(Y_{\theta }X_{\theta }^{T}\right)$ in the limit of infinite compositional input features: $n_{x}\to \infty$.

A consistent finding in the practical application of ML models is that their performance improves with the complexity of the task they are applied to. The study of these neural scaling laws has gained significant interest (77) and our present results would agree that increasing task complexity would push a neural network toward systematicity as the most efficient solution (78). Moreover, the distribution of word-use has been proposed to drive some of the most sophisticated and flexible behavior exhibited by Large Language Models (79). This distribution follows Zipf’s Law where the vast majority of words are seldom used, while a small set of words are used very frequently. *Observation* 7 seems consistent with this notion as we show that the addition of many objects or meanings to be expressed would push a network toward using the compositional structure of language out of necessity. Some work has also shown that compositionality can emerge within a single generation if multiple speakers are all contributing to the language (11). By rapidly increasing the number of input data points which need to be handled, sufficient tension between expressivity and regularity is imposed to form a compositional language, similar to IL. Thus, the inability of IL to ignore noncompositional input features can be mitigated by expanding the vocabulary of the language. The need for a sufficiently large meaning space for the emergence of systematicity has also been modeled and discussed in Nowak et al. (80).

### Generalizing Beyond the Space of Datasets.

In this section we have focused on analyzing IL in the space of datasets created by combining blocks of features with compositional and noncompositional structure. However, it is important to consider how our findings will generalize to other kinds of dataset structures. The space of datasets shows that when the high-rank, noncompositional features are appended to the low-rank, compositional features then the top singular vectors from the compositional features will rotate to accommodate some of the correlation introduced by the noncompositional features. In other words, the concepts in the language which are identifiable by the network change. As shown in Fig. 1 this means that no singular value corresponds to purely compositional input or output features. *Proposition* 1 shows that this effect of rotating singular vectors when appending different dataset structures does not depend on the substructures we have chosen being compositional and noncompositional. This demonstrates that our findings on systematicity do not depend on the space of datasets we mainly considered. The space of datasets does still formalize the notions of compositional and noncompositional substructure which is of primary interest when considering IL (8, 29, 81).

Proposition 1. Given a dataset (X,Y) removing an input or output feature cannot increase the first singular value of the input–output correlation matrix $\Sigma^{\mathrm{yx}}$.

**Proof Sketch**: We prove a generalization of the Cauchy Interlacing Theorem for nonsquare matrices which closely follows the strategy of Thompson (82). This shows that by considering any submatrix within a larger matrix the first singular value of the submatrix can only be equal or less than the first singular value of the original matrix. We apply this to the input–output correlation matrix $\Sigma^{\mathrm{yx}}$ to reach our conclusion.

## Discussion

We highlight one important question for future work: Do humans only possess the ability to generalize due to the vast number of concepts which we are exposed to in the world? This question is also important for machine learning, where the use of exceptionally large models has become the standard for achieving compositional or reasoning behaviors (83, 84). Of further interest is that our model predicts that generations of learners training on generated data can stabilize to a structured dataset. This is in contrast to recent findings on generative models which find that the recursive training of the models on previously generated data eventually leads to the degradation of model performance (85) (which has been termed “model collapse”). This has important implications for the longer-term utility of machine learning models, as generated data enters into subsequent models’ training pipelines (86). While our findings here provide a more hopeful outlook, extensions to our theoretical approach through the inclusion of nonlinearity and data which exists on low-dimensional manifolds (87) is necessary (we provide further discussion on the links between our findings and the machine learning and cognitive science literature in *SI Appendix*). In conclusion, we have shown the necessity of depth for artificial neural networks to enable IL to identify compositional substructure in language. This is due to the characteristics of the learning dynamics which occur with the addition of depth and are also found in children learning semantic information (38). The resultant language displays entangled compositionality (54) which contrasts with the typical structure imposed by modular neural networks, while still affording the same ease of systematic generalization when paired with a sufficiently large dataset. This strongly supports the use of IL as a contributing factor behind humans’ unique ability with language and provides a wealth of inspiration to direct future machine learning algorithms which aim to achieve systematic generalization.

## Materials and Methods

Our primary methodology revolves around the derivation of the closed-form learning dynamics for shallow and deep linear neural networks over generations of learning in the IL framework. This results in Eq. **3** for the shallow networks and Eq. **5** for the deep linear networks. These learning dynamics provide a change of basis for the neural networks from their parameters to their effective singular values. Prior work has shown that the dynamics of learning in terms of the singular values is reminiscent of a number of properties in child semantic learning when the network has hidden layers, but not when the network is shallow (38, 48). We leverage these properties of learning in deep linear networks to prove that depth is also necessary for IL to be an effective procedure to extract regularity from a dataset. Additionally, multiple generations of learners are necessary to guarantee that deep networks stabilize on the regular components of the dataset without losing expressivity. This corresponds to *Theorems* 1 and 2. Having established these general results for IL using the singular value dynamics of the neural networks, we then consider the consequences of these results for the emergence of compositionality in a dataset and the ability of the networks to systematically generalize. We formalize a space of datasets parameterized by five variables which determine the degree of compositional and noncompositional features on the input and output of the dataset (28). We obtain the input–output correlation matrix singular values for any dataset in the space, by writing the singular values in terms of these parameters, which allows us to make statements about how this dataset structure affects learning in the networks. From these dynamics, we find that IL is able to lower the rank on the input–output correlation matrix without losing expressivity in the dataset. Consequently, this creates a dataset which is easier to generalize on without losing the uniqueness of any of the data points. We define this as compositionality and the ability of the deep linear network to exploit this property of the dataset as systematic generalization, corresponding to *Observation* 6. We note however, that IL has no mechanism to deal with noncompositional input features and this still limits its ability to generalize. Thus, we present *Observation* 7 which shows that as the number of unique data points grows, the network will leverage compositional input features more and becomes systematic in the limit of infinite data.

## Supplementary Material

Appendix 01 (PDF)

## Data Availability

Code data have been deposited in https://github.com/CAandL-Lab/iterated_learning (60). All other data are included in the manuscript and/or *SI Appendix*.

## Footnotes

^*^All experiments are run using the Jax library (59). Full code for reproducing all figures is contained in *SI Appendix* and at: https://github.com/CAandL-Lab/iterated_learning (60).
