# Supplementary material for: Compositionality and systematicity emerge from iterated learning in deep linear networks
Source: Proc Natl Acad Sci U S A. 2026 May 5;123(19):e2509739123. doi: 10.1073/pnas.2509739123 (PMC13168545; doi:10.1073/pnas.2509739123)
Supplement: Supplementary file 1 — Appendix 01 (PDF) [file pnas.2509739123.sapp.pdf]

Supporting Information for

Compositionality and systematicity emerge from iterated learning in deep linear networks

Devon Jarvis, Richard Klein, Benjamin Rosman and Andrew M. Saxe

Corresponding Author: Devon Jarvis
E-mail: devon.jarvis@wits.ac.za

This PDF file includes:

Supporting text
Fig. S1
SI References

Supporting Information Text

Deep Linear Neural Network Training Dynamics Derivation

The theoretical strategy in this work is to calculate the training dynamics of linear neural networks or modules formed from these networks. A first important distinction is between deep and shallow linear networks. While deep linear networks can only represent linear input-output mappings, the dynamics of learning change dramatically with the introduction of one or more hidden layers (1–5), and the learning problem becomes non-convex (6). They therefore serve as a tractable model of the influence of depth specifically on learning dynamics, which prior work has shown to impart a low-rank inductive bias on the linear mapping (7). The exact solutions to the dynamics of learning from small random weights in deep linear networks have been derived in Saxe et al. (2014,2019) (2, 3) and as a result it is possible to obtain the full learning trajectory analytically for a number of representative tasks.

To review this paradigm consider a single-hidden-layer linear network with weight matrices W^1 and W^2 computing output $\hat{Y}_i = W^2 W^1 X_i$ where $\hat{Y}_i \in \mathbb{R}^D$, in response to an input data point $X_i \in \mathbb{R}^N$ from a dataset (X, Y) with P datapoints, and trained to minimize the quadratic loss using full batch gradient descent:

$$L(W^1, W^2) = \sum_{i=1}^{P} \frac{1}{2} ||Y_i - W^2 W^1 X_i||_2^2$$

This gives the learning rules for each layer with learning rate ϵ as:

$$\Delta W^1 = \epsilon \frac{\partial}{\partial W^1} L(W^1, W^2) = \sum_{i=1}^{P} \epsilon {W^2}^T (Y_i - W^2 W^1 X_i) X_i^T$$

$$\Delta W^2 = \epsilon \frac{\partial}{\partial W^2} L(W^1, W^2) = \sum_{i=1}^{P} \epsilon (Y_i - W^2 W^1 X_i)(W^1 X_i)^T$$

The average weight update across the dataset can be written in terms of the input correlation and input-output correlation matrices ($\frac{1}{P}\sum_{i=1}^{P} X_i X_i^T$ and $\frac{1}{P}\sum_{i=1}^{P} Y_i X_i^T$ respectively):

$$\begin{aligned}
\Delta W^1 &= \epsilon \frac{\partial}{\partial W^1} L(W^1, W^2) \\
&= \epsilon \sum_{i=1}^{P} {W^2}^T (Y_i - W^2 W^1 X_i) X_i^T \\
&= \epsilon P \frac{1}{P} \sum_{i=1}^{P} {W^2}^T (Y_i - W^2 W^1 X_i) X_i^T \\
&= \epsilon P {W^2}^T \left(\frac{1}{P} \sum_{i=1}^{P} Y_i X_i^T - W^2 W^1 \frac{1}{P} \sum_{i=1}^{P} X_i X_i^T \right) \\
&= \epsilon P {W^2}^T (\Sigma^{yx} - W^2 W^1 \Sigma^x) \\
\Delta W^2 &= \epsilon \frac{\partial}{\partial W^2} L(W^1, W^2) \\
&= \epsilon \sum_{i=1}^{P} (Y_i - W^2 W^1 X_i)(W^1 X_i)^T \\
&= \epsilon P \frac{1}{P} \sum_{i=1}^{P} (Y_i - W^2 W^1 X_i) X_i^T {W^1}^T \\
&= \epsilon P \left(\frac{1}{P} Y_i X_i^T - W^2 W^1 \frac{1}{P} X_i X_i^T \right) {W^1}^T \\
&= \epsilon P (\Sigma^{yx} - W^2 W^1 \Sigma^x) {W^1}^T
\end{aligned}$$

By using a small learning rate ϵ and taking the continuous time limit, the mean change in weights is given as:

$$\tau \frac{d}{dt} W^1 = {W^2}^T (\Sigma^{yx} - W^2 W^1 \Sigma^x); \qquad \tau \frac{d}{dt} W^2 = (\Sigma^{yx} - W^2 W^1 \Sigma^x) {W^1}^T$$

where $\tau = \frac{1}{P\epsilon}$ is the learning time constant. Here, t measures units of learning epochs. It is helpful to note that since we are using a small learning rate the full batch gradient descent and stochastic gradient descent dynamics will be the same.

Saxe et al. (2019) (3) has shown that the learning dynamics depend on the singular value decomposition of the correlation matrices. Let v^α denote the α-th singular vector of the square Σ^x matrix, and u^α denote the α-th left singular vector of Σ^{yx} with corresponding right singular vector v^α. Similarly, δ_α denotes a singular value of Σ^x and λ_α a singular value of Σ^{yx}. We collect the left singular vectors u^α into the matrix U and the right singular vectors v^α into V. The singular values can be collected into the diagonal matrices D and S for Σ^x and Σ^{yx} respectively. Finally if $rk(\cdot)$ denotes the rank of a matrix, then the SVDs are:

$$\Sigma^x = \sum_{\alpha=1}^{rk(\Sigma^x)} \delta_\alpha u^\alpha {v^\alpha}^T = USV^T; \qquad \Sigma^{yx} = \sum_{\alpha=1}^{rk(\Sigma^{yx})} \lambda_\alpha u^\alpha {v^\alpha}^T = VDV^T$$

To solve for the dynamics we require that Σ^{yx} and Σ^x are mutually diagonalizable such that the right singular vectors V of Σ^{yx} are also the singular vectors of Σ^x. We verify that this is true for the tasks considered in this work and assume it to be true for these derivations. We also assume that $n_h > rk(\Sigma^{yx})$ where n_h is the number of hidden neurons, so that the network can learn the desired mapping perfectly. If this is not the case then the network will only learn the top n_h singular values of the input-output mapping (2).

We now perform a change of variables using the SVD of the dataset statistics. The purpose of this step is to decouple the complex dynamics of the weights of the network, with interacting terms, into multiple one-dimensional systems. Specifically we set:

$$W^2 = U\overline{W}^2 R^T; \qquad W^1 = R\overline{W}^1 V^T$$

where R is an arbitrary orthogonal matrix such that $R^T R = I$. Substituting this into the gradient descent update rules for the parameters above yields:

$$\begin{aligned}
\tau \frac{d}{dt} W^1 &= {W^2}^T (\Sigma^{yx} - W^2 W^1 \Sigma^x) \\
\tau \frac{d}{dt} (R\overline{W}^1 V^T) &= R\overline{W}^2 U^T (USV^T - U\overline{W}^2 R^T R\overline{W}^1 V^T VDV^T) \\
\tau \frac{d}{dt} (R\overline{W}^1 V^T) &= R\overline{W}^2 (SV^T - \overline{W}^2 \overline{W}^1 DV^T) \\
\tau \frac{d}{dt} \overline{W}^1 &= \overline{W}^2 (S - \overline{W}^2 \overline{W}^1 D)
\end{aligned}$$

and

$$\begin{aligned}
\tau \frac{d}{dt} W^2 &= (\Sigma^{yx} - W^2 W^1 \Sigma^x) {W^1}^T \\
\tau \frac{d}{dt} (U\overline{W}^2 R^T) &= (USV^T - U\overline{W}^2 R^T R\overline{W}^1 V^T VDV^T) V\overline{W}^1 R^T \\
\tau \frac{d}{dt} (U\overline{W}^2 R^T) &= (US - U\overline{W}^2 \overline{W}^1 D) \overline{W}^1 R^T \\
\tau \frac{d}{dt} \overline{W}^2 &= \overline{W}^1 (S - \overline{W}^2 \overline{W}^1 D)
\end{aligned}$$

Here we have used the orthogonality of the singular vectors such that $V^T V = I$ and $U^T U = I$. Importantly, if the assumptions that the weight matrices align and the correlation matrices are mutually diagonalizable hold, then all matrices in the dynamics are now diagonal and represent the decoupling of the network into the modes transmitted from input to the hidden neurons and from hidden to output neurons. In practice we do not initialize the network weights to adhere to this diagonalization and so it is not guaranteed that the matrices will be diagonal at initialization. However, empirically it has been found that the network singular vectors rapidly align to this required configuration (2, 3) and this has been termed the "silent alignment effect" (8). However, if this alignment does not occur fully then the weight matrices after the change of variables will not be diagonal. The alignment assumption corresponds to the assumption that the network is feature learning perfectly. While the need for perfect feature learning is strong, it is a valid start for the questions around feature learning considered in this work. If the assumption of the correlation matrices being mutually diagonalizable does not hold then the update equations simplify to:

$$\tau \frac{d}{dt} \overline{W}^1 = \overline{W}^2 (S\hat{V}^T V - \overline{W}^2 \overline{W}^1 D)$$

and

$$\tau \frac{d}{dt} \overline{W}^2 = \overline{W}^1 (S\hat{V}^T V - \overline{W}^2 \overline{W}^1 D)$$

where $\hat{V}$ now denotes the right singular vectors of Σ^{yx} and are different from V from Σ^x. We note that the remainder of the derivation requires both assumptions, however these two equations provide a valid dynamics reduction which could be sufficient for many interesting cases. If the weight alignment holds then this is especially true as $\hat{V}^T V$ would become an small

interpretable matrix used to align the singular vectors from the correlation matrices. Letting ω^1_α be the α-th mode of $\overline{W}^1$ and ω^2_α be the α-th mode of $\overline{W}^2$ we can write the individual mode dynamics as:

$$\tau \frac{d}{dt}\omega^1_\alpha = \omega^2_\alpha(\lambda_\alpha - \omega^2_\alpha\omega^1_\alpha\delta_\alpha); \qquad \tau \frac{d}{dt}\omega^2_\alpha = \omega^1_\alpha(\lambda_\alpha - \omega^2_\alpha\omega^1_\alpha\delta_\alpha)$$

In general ω^1_α and ω^2_α can be different but if they are initialized with small values then they will be roughly equal. We study this balanced setting and assume it to be true for all dynamics calculations in this work. Thus we let $\omega^1_\alpha = \omega^2_\alpha$ and track the dynamics of an entire mode as $\pi_\alpha = \omega^2_\alpha\omega^1_\alpha$. Using the product rules this gives the separable differential equation (we will drop the dependence on α for now for notational convenience):

$$\begin{aligned}
\tau \frac{d}{dt}\pi =& \omega^1(\tau\frac{d}{dt}\omega^2) + (\tau\frac{d}{dt}\omega^1)\omega^2 \\
\tau \frac{d}{dt}\pi =& \omega^1(\omega^1(\lambda - \omega^2\omega^1\delta)) + (\omega^2(\lambda - \omega^2\omega^1\delta))\omega^2 \\
\tau \frac{d}{dt}\pi =& (\omega^1)^2(\lambda - \omega^2\omega^1\delta) + (\omega^2)^2(\lambda - \omega^2\omega^1\delta) \\
\tau \frac{d}{dt}\pi =& \pi(\lambda - \pi\delta) + \pi(\lambda - \pi\delta) \\
\tau \frac{d}{dt}\pi =& 2\pi(\lambda - \pi\delta)
\end{aligned}$$

Integrating to solve for t then yields:

$$\begin{aligned}
dt =& \frac{\tau}{2}\frac{d\pi}{\pi(\lambda - \pi\delta)} \\
t =& \frac{\tau}{2}\int_{\pi_0}^{\pi_f}\frac{d\pi}{\pi(\lambda - \pi\delta)} \\
t =& \frac{\tau}{2\lambda} ln\frac{\pi_f(\lambda - \pi_0\delta)}{\pi_0(\lambda - \pi_f\delta)}
\end{aligned}$$

where t is the time taken for the mode to reach a value $\pi(t) = \pi_f$ from the initial strength $\pi(0) = \pi_0$. By re-arranging the terms we can obtain the dynamics of the mode for all points in time:

$$\pi_\alpha(t) = \frac{\lambda_\alpha/\delta_\alpha}{1 - (1 - \frac{\lambda_\alpha}{\delta_\alpha\pi_0})\exp(\frac{-2\lambda_\alpha}{\tau}t)} \qquad [1]$$

All together this means that, given the SVDs of the two correlation matrices, the learning dynamics can be described explicitly by setting $A(t)$ as the effective singular value matrix of the network's mapping and the trajectory of each singular value in $A(t)$ is described by $\pi_\alpha(t)$. With the assumption of the network singular vectors aligning to the dataset singular vectors, these effective singular values begin at the initial value π_0 when $t = 0$ and increases to $\pi^*_\alpha = \lambda_\alpha/\delta_\alpha$ as $t \to \infty$:

$$W^2(t)W^1(t) = UA(t)V^T = \sum_{\alpha=1}^{rk(\Sigma^{yx})}\pi_\alpha(t)u^\alpha v^{\alpha T}$$

We note that for the space of datasets the rank of the input-output correlation matrix for an initial dataset will be $rk(\Sigma^{yx}) = 2^{n_x}$. From the dynamics equation shown in Equation 1, it is helpful to note that the time-course of the trajectory is only dependent on the Σ^{yx} singular values. Thus, Σ^x affects the stable point of the network singular values but not the time-course of learning.

Shallow Linear Neural Network Training Dynamics Derivation

To review the shallow neural network paradigm consider a linear network with weight matrix W computing output $\hat{Y}_i = WX_i$ where $\hat{Y}_i \in \mathbb{R}^D$, in response to an input data point $X_i \in \mathbb{R}^N$ from a dataset (X, Y) with P datapoints, and trained to minimize the quadratic loss using full batch gradient descent:

$$L(W) = \sum_{i=1}^{P}\frac{1}{2}||Y_i - WX_i||^2_2$$

This gives the learning rules with learning rate ϵ as:

$$\Delta W = \frac{\partial}{\partial W}L(W) = \sum_{i=1}^{P}\epsilon(Y_i - WX_i)X_i^T$$

The average weight update across the dataset can be written in terms of the input correlation and input-output correlation matrices ($\frac{1}{P}\sum_{i=1}^{P} X_i X_i^T$ and $\frac{1}{P}\sum_{i=1}^{P} Y_i X_i^T$ respectively):

$$\begin{aligned}\Delta W &= \epsilon \frac{\partial}{\partial W} L(W) = \epsilon \sum_{i=1}^{P} (Y_i - W X_i) X_i^T \\ &= \epsilon P \frac{1}{P} \sum_{i=1}^{P} (Y_i - W X_i) X_i^T \\ &= \epsilon P \left(\frac{1}{P} \sum_{i=1}^{P} Y_i X_i^T - W \frac{1}{P} \sum_{i=1}^{P} X_i X_i^T \right) \\ &= \epsilon P (\Sigma^{yx} - W \Sigma^{x})\end{aligned}$$

By using a small learning rate ϵ and taking the continuous time limit, the mean change in weights is given as:

$$\tau \frac{d}{dt} W = \Sigma^{yx} - W \Sigma^{x}$$

where $\tau = \frac{1}{P\epsilon}$ is the learning time constant. Here, t measures units of learning epochs. It is helpful to note that since we are using a small learning rate the full batch gradient descent and stochastic gradient descent dynamics will be the same. Saxe et al. (2019) (3) has shown that the learning dynamics depend on the singular value decomposition of the correlation matrices. Let v^{α} denote the α-th singular vector of the square Σ^{x} matrix, and u^{α} denote the α-th left singular vector of Σ^{yx} with corresponding right singular vector v^{α}. Similarly, δ_{α} denotes a singular value of Σ^{x} and λ_{α} a singular value of Σ^{yx}. We collect the left singular vectors u^{α} into the matrix U and the right singular vectors v^{α} into V. The singular values can be collected into the diagonal matrices D and S for Σ^{x} and Σ^{yx} respectively. Finally if $rk(\cdot)$ denotes the rank of a matrix, then the SVDs are:

$$\Sigma^{x} = \sum_{\alpha=1}^{rk(\Sigma^{x})} \delta_{\alpha} u^{\alpha} {v^{\alpha}}^T = USV^T; \qquad \Sigma^{yx} = \sum_{\alpha=1}^{rk(\Sigma^{yx})} \lambda_{\alpha} u^{\alpha} {v^{\alpha}}^T = VDV^T$$

To solve for the dynamics we require that Σ^{yx} and Σ^{x} are mutually diagonalizable such that the right singular vectors V of Σ^{yx} are also the singular vectors of Σ^{x}. We verify that this is true for the tasks considered in this work and assume it to be true for these derivations. We now perform a change of variables using the SVD of the dataset statistics. The purpose of this step is to decouple the complex dynamics of the weights of the network, with interacting terms, into multiple one-dimensional systems. Specifically we set:

$$W = U\overline{W}V^T$$

Substituting this into the gradient descent update rules for the parameters above yields:

$$\begin{aligned}\tau \frac{d}{dt} W &= \Sigma^{yx} - W\Sigma^{x} \\ \tau \frac{d}{dt} (U\overline{W}V^T) &= USV^T - U\overline{W}V^T VDV^T \\ \tau \frac{d}{dt} (U\overline{W}V^T) &= USV^T - U\overline{W}DV^T \\ \tau \frac{d}{dt} \overline{W} &= S - \overline{W}D\end{aligned}$$

Here we have used the orthogonality of the singular vectors such that $V^T V = I$ and $U^T U = I$. Importantly, if the assumptions that the weight matrices align and the correlation matrices are mutually diagonalizable hold, then all matrices in the dynamics are now diagonal and represent the decoupling of the network into the modes transmitted from input to output neurons. In practice we do not initialize the network weights to adhere to this diagonalization and so it is not guaranteed that the matrices will be diagonal at initialization. However, empirically it has been found that the network singular vectors rapidly align to this required configuration (2, 3) and this has been termed the "silent alignment effect" (8). However, if this alignment does not occur fully then the weight matrices after the change of variables will not be diagonal. The alignment assumption corresponds to the assumption that the network is feature learning perfectly. While the need for perfect feature learning is strong, it is a valid start for the questions around feature learning considered in this work. Letting π_{α} be the α-th mode of $\overline{W}$ we can write the individual mode dynamics as:

$$\tau \frac{d}{dt} \pi_{\alpha} = \lambda_{\alpha} - \pi_{\alpha}\delta_{\alpha}$$

We now drop the dependence on α for notational convenience. Integrating to solve for t then yields:

$$
\begin{aligned}
dt =& \tau \frac{d\pi}{\lambda - \pi\delta} \\
t =& \tau \int_{\pi_0}^{\pi_f} \frac{d\pi}{\lambda - \pi\delta} \\
t =& \frac{\tau}{\delta} \ln \frac{\lambda - \pi^0 \delta}{\lambda - \pi^f \delta}
\end{aligned}
$$

where t is the time taken for the mode to reach a value $\pi(t) = \pi_f$ from the initial strength $\pi(0) = \pi_0$. By re-arranging the terms we can obtain the dynamics of the mode for all points in time:

$$
\pi_\alpha(t) = \frac{\lambda_\alpha}{\delta_\alpha} \left(1 - \exp(-\delta_\alpha t/\tau)\right) + \pi_\alpha^0 \exp(-\delta_\alpha t/\tau) \qquad [2]
$$

All together this means that, given the SVDs of the two correlation matrices, the learning dynamics can be described explicitly by setting $A(t)$ as the effective singular value matrix of the network's mapping and the trajectory of each singular value in $A(t)$ is described by $\pi_\alpha(t)$. With the assumption of the network singular vectors aligning to the dataset singular vectors, these effective singular values begin at the initial value π_0 when $t = 0$ and increases to $\pi_\alpha^* = \lambda_\alpha/\delta_\alpha$ as $t \to \infty$:

$$
W(t) = UA(t)V^T = \sum_{\alpha=1}^{rk(\Sigma^{yx})} \pi_\alpha(t) u^\alpha v^{\alpha T}
$$

Derivation of Hitting and Convergence Time

We will begin by using the definition of hitting time and substituting the mode dynamics (Equation 4) into this expression to obtain an expression for t:

$$
\begin{aligned}
\pi_\alpha^G =& \rho \\
\frac{\lambda_\alpha^G/\delta_\alpha}{1 - \left(1 - \frac{\lambda_\alpha^G}{\delta_\alpha \pi_0}\right) \exp\left(\frac{-2\lambda_\alpha^G}{\tau} t\right)} =& \rho \\
\frac{\lambda_\alpha^G/\delta_\alpha}{\rho} =& 1 - \left(1 - \frac{\lambda_\alpha^G}{\delta_\alpha \pi_0}\right) \exp\left(\frac{-2\lambda_\alpha^G}{\tau} t\right) \\
\frac{\rho - \lambda_\alpha^G/\delta_\alpha}{\rho} =& \left(1 - \frac{\lambda_\alpha^G}{\delta_\alpha \pi_0}\right) \exp\left(\frac{-2\lambda_\alpha^G}{\tau} t\right) \\
\frac{\rho - \lambda_\alpha^G/\delta_\alpha}{\rho\left(1 - \frac{\lambda_\alpha^G}{\delta_\alpha \pi_0}\right)} =& \exp\left(\frac{-2\lambda_\alpha^G}{\tau} t\right) \\
\log\left(\frac{\rho - \lambda_\alpha^G/\delta_\alpha}{\rho\left(1 - \frac{\lambda_\alpha^G}{\delta_\alpha \pi_0}\right)}\right) =& \frac{-2\lambda_\alpha^G}{\tau} t \\
\frac{-\tau}{2\lambda_\alpha^G} \log\left(\frac{\rho - \lambda_\alpha^G/\delta_\alpha}{\rho\left(1 - \frac{\lambda_\alpha^G}{\delta_\alpha \pi_0}\right)}\right) =& t \\
\frac{-\tau}{2\lambda_\alpha^G} \log\left(\frac{\rho\left(1 - \frac{\lambda_\alpha^G}{\delta_\alpha \rho}\right)}{\rho\left(1 - \frac{\lambda_\alpha^G}{\delta_\alpha \pi_0}\right)}\right) =& t \\
\frac{\tau}{2\lambda_\alpha^G} \log\left(\frac{1 - \frac{\lambda_\alpha^G}{\delta_\alpha \pi_0}}{1 - \frac{\lambda_\alpha^G}{\delta_\alpha \rho}}\right) =& t \\
\frac{\tau}{2\lambda_\alpha^G} \log\left(\frac{\frac{\lambda_\alpha^G}{\delta_\alpha \pi_0} - 1}{\frac{\lambda_\alpha^G}{\delta_\alpha \rho} - 1}\right) =& t \\
\frac{\tau}{2\lambda_\alpha^G} \log\left(\frac{\frac{\lambda_\alpha^G - \delta_\alpha \pi_0}{\delta_\alpha \pi_0}}{\frac{\lambda_\alpha^G - \delta_\alpha \rho}{\delta_\alpha \rho}}\right) =& t
\end{aligned}
$$

$$
\begin{aligned}
\frac{\tau}{2\lambda_\alpha^G}\log\left(\frac{\lambda_\alpha^G\delta_\alpha\rho-\delta_\alpha^2\pi_0\rho}{\lambda_\alpha^G\delta_\alpha\pi_0-\delta_\alpha^2\pi_0\rho}\right) &= t \\
\frac{\tau}{2\lambda_\alpha^G}\log\left(\frac{\lambda_\alpha^G\rho-\delta_\alpha\pi_0\rho}{\lambda_\alpha^G\pi_0-\delta_\alpha\pi_0\rho}\right) &= t
\end{aligned}
$$

The term inside of the log is always going to be greater than 1 as $\rho \geq \pi_0$. Thus the log is positive. In the extreme case where $\rho = \pi_0$ then the log evaluates to 0 as the internal fraction is 1, which makes sense as in this case the hitting time will be reached at initialization. We can perform a similar computation for a modes convergence time.

$$
\begin{aligned}
(\lambda_\alpha^G/\delta_\alpha)-\pi_\alpha^G &= \rho \\
(\lambda_\alpha^G/\delta_\alpha)-\frac{\lambda_\alpha^G/\delta_\alpha}{1-\left(1-\frac{\lambda_\alpha^G}{\delta_\alpha\pi_0}\right)\exp\left(\frac{-2\lambda_\alpha^G}{\tau}t\right)} &= \rho \\
-\frac{\lambda_\alpha^G/\delta_\alpha}{1-\left(1-\frac{\lambda_\alpha^G}{\delta_\alpha\pi_0}\right)\exp\left(\frac{-2\lambda_\alpha^G}{\tau}t\right)} &= \rho-(\lambda_\alpha^G/\delta_\alpha) \\
-\frac{\lambda_\alpha^G/\delta_\alpha}{\rho-(\lambda_\alpha^G/\delta_\alpha)} &= 1-\left(1-\frac{\lambda_\alpha^G}{\delta_\alpha\pi_0}\right)\exp\left(\frac{-2\lambda_\alpha^G}{\tau}t\right) \\
-\frac{\lambda_\alpha^G/\delta_\alpha}{\rho-(\lambda_\alpha^G/\delta_\alpha)}-\frac{\rho-(\lambda_\alpha^G/\delta_\alpha)}{\rho-(\lambda_\alpha^G/\delta_\alpha)} &= -\left(1-\frac{\lambda_\alpha^G}{\delta_\alpha\pi_0}\right)\exp\left(\frac{-2\lambda_\alpha^G}{\tau}t\right) \\
\frac{\rho}{\rho-(\lambda_\alpha^G/\delta_\alpha)} &= \left(1-\frac{\lambda_\alpha^G}{\delta_\alpha\pi_0}\right)\exp\left(\frac{-2\lambda_\alpha^G}{\tau}t\right) \\
\frac{\rho}{(\rho-(\lambda_\alpha^G/\delta_\alpha))\left(1-\frac{\lambda_\alpha^G}{\delta_\alpha\pi_0}\right)} &= \exp\left(\frac{-2\lambda_\alpha^G}{\tau}t\right) \\
\log\left(\frac{\rho}{(\rho-(\lambda_\alpha^G/\delta_\alpha))\left(1-\frac{\lambda_\alpha^G}{\delta_\alpha\pi_0}\right)}\right) &= \frac{-2\lambda_\alpha^G}{\tau}t \\
\frac{-\tau}{2\lambda_\alpha^G}\log\left(\frac{\rho}{((\lambda_\alpha^G/\delta_\alpha)-\rho)\left(\frac{\lambda_\alpha^G}{\delta_\alpha\pi_0}-1\right)}\right) &= t \\
\frac{\tau}{2\lambda_\alpha^G}\log\left(\frac{((\lambda_\alpha^G/\delta_\alpha)-\rho)\left(\frac{\lambda_\alpha^G}{\delta_\alpha\pi_0}-1\right)}{\rho}\right) &= t \\
\frac{\tau}{2\lambda_\alpha^G}\log\left(\frac{\lambda_\alpha^{G^2}}{\delta_\alpha^2\pi_0\rho}-\frac{\lambda_\alpha^G}{\delta_\alpha\rho}-\frac{\lambda_\alpha^G}{\delta_\alpha\pi_0}+1\right) &= t \\
\frac{\tau}{2\lambda_\alpha^G}\log\left(\frac{\lambda_\alpha^{G^2}-\delta_\alpha\lambda_\alpha^G\pi_0-\delta_\alpha\lambda_\alpha^G\rho+\delta_\alpha^2\pi_0\rho}{\delta_\alpha^2\pi_0\rho}\right) &= t
\end{aligned}
$$

Thus we can summarize the hitting time and convergence time for mode α as follows:

$$
\hat{t}_\alpha = \frac{\tau}{2\lambda_\alpha^G}\log\left(\frac{\lambda_\alpha^G\rho-\delta_\alpha\pi_0\rho}{\lambda_\alpha^G\pi_0-\delta_\alpha\pi_0\rho}\right);\; t_\alpha^* = \frac{\tau}{2\lambda_\alpha^G}\log\left(\frac{\lambda_\alpha^{G^2}-\delta_\alpha\lambda_\alpha^G\pi_0-\delta_\alpha\lambda_\alpha^G\rho+\delta_\alpha^2\pi_0\rho}{\delta_\alpha^2\pi_0\rho}\right)
$$

Singular Value Decomposition and Frobenius Norm Equations

In this section we provide the general formulas for the singular value decomposition of the Σ^x and Σ^{yx} covariance matrices for any dataset in the space of datasets. As stated in the main text the right singular vectors of Σ^{yx} must match the singular vectors of Σ^x which is the V matrix below. Thus, $\Sigma^x = VDV^T$ and $\Sigma^{yx} = USV^T$.

Let:
$A = (Y_\Omega X_\Omega^T)^T Y_\Omega X_\Omega^T$
$B = Y_\Omega^T Y_\Omega X_\Omega^T$
$C = X_\Omega^T X_\Omega X_\Omega^T$

$THP^T = (\frac{1}{k_x k_y})^{\frac{1}{2}} \mathbf{I_{2^{n_x} \times 2^{n_x}}} - (\frac{1}{k_x k_y})^{\frac{1}{2}} (\frac{1}{2^{n_x}}) X_\Omega^T X_\Omega$

Where THP^T is the SVD of $(\frac{1}{k_x k_y})^{\frac{1}{2}} \mathbf{I_{2^{n_x} \times 2^{n_x}}} - (\frac{1}{k_x k_y})^{\frac{1}{2}} (\frac{1}{2^{n_x}}) X_\Omega^T X_\Omega$, and $H = (\frac{1}{k_x k_y})^{\frac{1}{2}} \mathbf{I_{2^{n_x} \times 2^{n_x}}}$. Then the following are the matrix formulas for the components of the SVD for Σ^{yx} and Σ^x.

$$U = \begin{bmatrix} \left(\frac{1}{2^{n_x}(k_y r^2 + 2^{n_x})}\right)^{\frac{1}{2}} Y_\Omega X_\Omega^T & \mathbf{0_{n_y \times k_x 2^{n_x}}} \\ \left(\frac{r^2}{2^{3n_x}(k_y r^2 + 2^{n_x})}\right)^{\frac{1}{2}} B + \left(\frac{r^2}{2^{3n_x}(k_y r^2)}\right)^{\frac{1}{2}} (C - B) & (\frac{1}{k_y})^{\frac{1}{2}} T \end{bmatrix} \quad [3]$$

$$V^T = \begin{bmatrix} \left(\frac{2^{n_x}}{(k_x r^2 + 2^{n_x})}\right)^{\frac{1}{2}} \mathbf{I_{n_x \times n_x}} & \left(\frac{r^2}{2^{n_x}(k_x r^2 + 2^{n_x})}\right)^{\frac{1}{2}} X_\Omega \\ \mathbf{0_{k_x 2^{n_x} \times n_x}} & (\frac{1}{k_x})^{\frac{1}{2}} P^T \end{bmatrix} \quad [4]$$

$$S = \begin{bmatrix} \left(\frac{(k_x r^2 + 2^{n_x})(k_y r^2 + 2^{n_x})}{2^{6n_x}}\right)^{\frac{1}{2}} A + \left(\frac{(k_x r^2 + 2^{n_x})(k_y r^2)}{2^{2n_x}}\right)^{\frac{1}{2}} (I - \frac{1}{2^{2n_x}} A) & \mathbf{0_{n_x \times k_x 2^{n_x}}} \\ \mathbf{0_{k_x 2^{n_x} \times n_x}} & (k_x k_y)^{\frac{1}{2}} \frac{r^2}{2^{n_x}} \mathbf{I_{k_x 2^{n_x} \times k_x 2^{n_x}}} \end{bmatrix} \quad [5]$$

$$D = \begin{bmatrix} \left(\frac{(k_x r^2 + 2^{n_x})}{2^{n_x}}\right)^{\frac{1}{2}} \mathbf{I_{n_x \times n_x}} & \mathbf{0_{n_x \times k_x 2^{n_x}}} \\ \mathbf{0_{k_x 2^{n_x} \times n_x}} & \frac{k_x r^2}{2^{n_x}} \mathbf{I_{2^{n_x} \times 2^{n_x}}} \end{bmatrix} \quad [6]$$

We note that each distinct singular value occurs multiple times in the dataset: λ_1 has multiplicity $n_x - n_y$, λ_2 has multiplicity n_y, and λ_3 has multiplicity $2^{n_x} - n_x$. Correspondingly δ_1 has multiplicity n_x and δ_2 has multiplicity $2^{n_x} - n_x$.

Utilizing this SVD and the closed-form learning dynamics they afford, we also consider the Frobenius norms of portions of the network mapping connecting the four main dataset substructures. Specifically, we determine the degree of association between substructures by first taking the product of the network weights ($W^* = W_2 W_1$), which is possible since matrix multiplication is associative and there are no activation functions. We then calculate the Frobenius norm of substructures of W^* connected to the relevant input and output substructures. For example, if we aim to measure the association between compositional input ($X_\Omega = X_{[0:2^{n_x}]}$) and non-compositional output ($Y_\Gamma = Y_{[2^{n_y}:2^{n_y}+k_y 2^{n_x}]}$) then we would calculate the Frobenius norm of $W^*_{[2^{n_y}:2^{n_x}+k_y 2^{n_x}, 0:2^{n_x}]}$. The norms depicting the relation between the two input substructures with the output substructures (four in total) for multiple generations of learning are shown in Equations 7-10.

$$X_\Omega Y_\Omega^G\text{-Norm} = \left(\frac{2^{2n_x} n_y \pi_1^2(t)}{(k_x r^2 + 2^{n_x})(k_y r^2 + 2^{n_x})}\right)^{\frac{1}{2}} \quad [7] \qquad X_\Gamma Y_\Omega^G\text{-Norm} = \left(\frac{2^{n_x} n_y k_x r^2 \pi_1^2(t)}{(k_x r^2 + 2^{n_x})(k_y r^2 + 2^{n_x})}\right)^{\frac{1}{2}} \quad [8]$$

$$X_\Omega Y_\Gamma^G\text{-Norm} = \left(\frac{2^{n_x} k_y n_y r^2 \pi_1^2(t)}{(k_x r^2 + 2^{n_x})(k_y r^2 + 2^{n_x})} + \frac{2^{n_x}(n_x - n_y)}{k_x r^2 + 2^{n_x}} \pi_2^2(t)\right)^{\frac{1}{2}} \quad [9]$$

$$X_\Gamma Y_\Gamma^G\text{-Norm} = \left(\frac{k_x k_y n_y r^4 \pi_1^2(t)}{(k_x r^2 + 2^{n_x})(k_y r^2 + 2^{n_x})} + \frac{(n_x - n_y) k_x r^2}{k_x r^2 + 2^{n_x}} \pi_2^2(t) + (2^{n_x} - n_x)\pi_3^2(t)\right)^{\frac{1}{2}} \quad [10]$$

Pairing Modularity and Iterated Learning

One of the primary reasons shown in prior work (9) to use modularity is that it is able to completely separate compositional substructure in a dataset. This allows a network to leverage the low-rank nature of the compositional substructure to generalize in this portion of the output. To make this point explicit in our setup, we present Observation 0.1 which proves that IL with a densely connected network is unable to completely refine the output language and produce a compositional language for all datasets in the space. Note the distinction between compositional and entangled compositional language in this case. As we show in the main text, IL is able to identify low-rank substructure which is entangled compositional and supports systematic generalization.

Observation 0.1. *For all points in the space of datasets: $n_x, n_y, k_x, k_y, r \in \mathbb{Z}^+$ with the dense network architecture: $X_\Omega Y_\Omega^G$-Norm will reach its escaping time in the same time course as $X_\Omega Y_\Gamma^G$-Norm and $X_\Gamma Y_\Gamma^G$-Norm $\forall\ G > 0$.*

Proof Sketch: We begin by noting that the escaping time of a Frobenius norm is the minimum of the escaping times of the modes which contribute to the norm. Thus, to prove this result we prove that of the three kinds of modes ($\pi_1(t)$, $\pi_2(t)$ and $\pi_3(t)$) the $\pi_1(t)$ mode has the minimum escaping time. We then note that all three Frobenius norms considered ($X_\Omega Y_\Omega^G$-Norm, $X_\Omega Y_\Gamma^G$-Norm and $X_\Gamma Y_\Gamma^G$-Norm) have some contribution from this mode. As a consequence all three norms have the same escaping time.

The consequence of Observation 0.1 is that there will always be a portion of the network mapping producing non-compositional output substructure. To obtain a purely compositional mapping on the space of datasets prior work showed that a modular network architecture is required which **perfectly** partitions the compositional and non-compositional input and output substructure before learning even begins (9). By partitioning substructure in this manner each module will be learning on a special case of the original dataset. For example, the module connecting only compositional input and output will be learning on an effective dataset with $k_x = k_y = 0$. As a result, this will change the training dynamics of each module. If we consider the weaker architectural prior of a perfect partition of output substructure the network Frobenius norms simplify to Equations 11-14.

$$X_\Omega Y_\Omega^G\text{-Norm} = \left(\frac{2^{n_x} n_y}{k_x r^2 + 2^{n_x}} \pi_1^{G^2}(t)\right)^{\frac{1}{2}} \qquad [11]$$

$$X_\Gamma Y_\Omega^G\text{-Norm} = \left(\frac{n_y k_x r^2}{k_x r^2 + 2^{n_x}} \pi_1^{G^2}(t)\right)^{\frac{1}{2}} \qquad [12]$$

$$X_\Omega Y_\Gamma^G\text{-Norm} = \left(\frac{2^{n_x} n_x}{k_x r^2 + 2^{n_x}} \pi_2^{G^2}(t)\right)^{\frac{1}{2}} \qquad [13]$$

$$X_\Gamma Y_\Gamma^G\text{-Norm} = \left(\frac{n_x k_x r^2}{k_x r^2 + 2^{n_x}} \pi_2^{G^2}(t) + (2^{n_x} - n_x)\pi_3^{G^2}(t)\right)^{\frac{1}{2}} \qquad [14]$$

With the modular norm equations we now make Observation 0.2. The consequence of this observation is that, with the architectural bias of perfect output partitioning, IL is able to produce a compositional language. This is due to the π_1 effective singular value now only being responsible for producing compositional output (appearing in the $X_\Omega Y_\Omega^G$-Norm and $X_\Gamma Y_\Omega^G$-Norm only) allowing for different output substructure to begin learning at different times.

Observation 0.2. *For all points in the space of datasets: $n_x, n_y, k_x, k_y, r \in \mathbb{Z}^+$ with the output-partitioned network architecture: $X_\Omega Y_\Omega^G$-Norm and $X_\Gamma Y_\Omega^G$-Norm will reach their hitting time before $X_\Omega Y_\Gamma^G$-Norm and $X_\Gamma Y_\Gamma^G$-Norm reach their escaping time for some $G > 0$.*

Proof Sketch: We note that for the Frobenius norms their escaping time is equal to the minimum escaping time of all modes which contribute to it and their hitting time is equal to the maximum hitting time of all the modes which contribute to it. Thus, in the split network the $X_\Omega Y_\Omega^G$-Norm and $X_\Gamma Y_\Omega^G$-Norm have a hitting time of t_1^*. The escaping time of $X_\Gamma Y_\Gamma^G$-Norm is $\hat{t}_2$ and for $X_\Gamma Y_\Gamma^G$-Norm the escaping time is $\hat{t}_3$. From Observation 1 we know that the modes will be ordered as $\lambda_1 > \lambda_2 > \lambda_3$. Thus, due to Theorem 2 there is a generation $G > 0$ for which we are guaranteed to have $\hat{t}_2, \hat{t}_3 > t_1^*$. Thus, there is some $G > 0$ for which the hitting time of $X_\Omega Y_\Omega^G$-Norm and $X_\Gamma Y_\Omega^G$-Norm is less than the escaping time of $X_\Omega Y_\Gamma^G$-Norm and $X_\Gamma Y_\Gamma^G$-Norm.

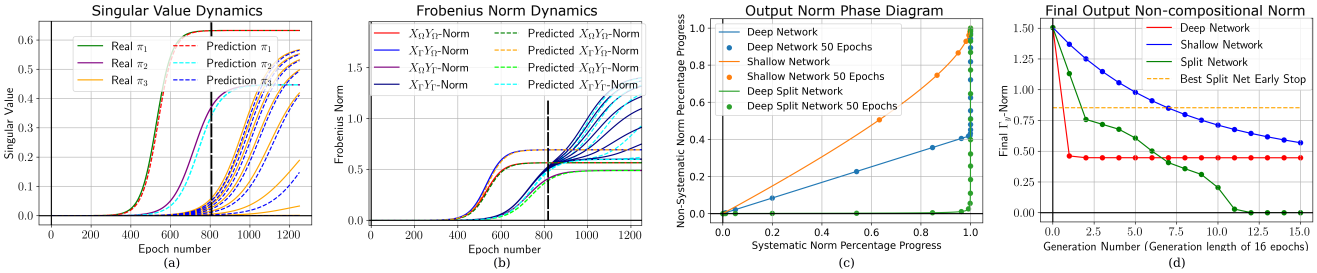

Fig. S1. The need for modularity with IL for compositional language to emerge: (a) Comparison of the modular network's predicted and actual singular value trajectories over learning, for the three unique dataset singular values. (b) Comparison of predicted and actual Frobenius norms of the input-output mapping to/from systematic (X_Ω, Y_Ω) and non-systematic (X_Γ, Y_Γ) features for the modular network. Compared to Figure 2(b) we now see that the two norms connecting to the non-compositional output begin learning at different times to those connecting to the compositional output. In contrast, all norms for the dense network (Figure 2(b)) begin learning at the same time, when the first effective singular value π_1 is learned. Consequently, IL is now able to exploit this modularity to completely remove the non-compositional output mapping if the correct early stopping time is chosen. This time is depicted by the vertical black line in (a) and (b) (we only demonstrate the removal of one mode in the figure, but removing both π_2 and π_3 is necessary to remove the non-compositional output). (c) Phase diagram depicting the relative speed with which different architectures learn the compositional and non-compositional output mapping for a final generation (in other words a generation where no further refinement of the language can occur). Shallow networks learn all information at nearly the same time, deep networks learn the complete compositional output first, but still learn some non-compositional output before this, limiting the benefit of IL. The modular network learns all of the compositional outputs (reaches its hitting time) before the non-compositional output begins to learn (reaches its escaping time). Thus, IL is able to completely remove the non-compositional output. We note that all that is required is to have some $G > 0$, it does not need to be for $G = 1$. (d) The Frobenius norm of the network mapping to non-compositional output (using all inputs) over 15 generations. The shallow network is unable to reduce any non-compositional information without also losing compositional information (which is not allowed but we still show some refinement here). The dense network removes most of the non-compositional output information but is unable to remove all of it. The modular network is able to remove all non-compositional outputs and create a compositional language. We compare to the optimal early stopping time (shown in yellow) for the split network and see that early stopping would be unable to completely remove the non-compositional portion of the language, as we prove in Observation 2. *Dataset Parameters*: $n_x = 3, n_y = 2, k_x = 3, k_y = 1, r = 2$.

Thus, when paired with architectural modularity IL is able to produce a purely compositional language. We demonstrate this empirically in Figure S1 by tracking the singular value and Frobenius norm dynamics of the split network. From a neuroscientific perspective this also presents an interesting result, as it implies that the compositional nature of natural language is influenced by architectural biases towards forming specialized regions performing modular roles within a larger computation. Thus, our result is consistent with experimental studies which have found specialized brain regions (by establishing double dissociation) in the production of different word classes such as nouns or adverbs (10–14).

Extended Discussion

From a purely linguistics perspective we demonstrate that multiple generations of learners is required to guarantee that a language will be refined to be as easily learnable as possible. In this case the ease of learning stems from the lower-rank structure of the emergent language which supports generalization. We then find that this does not require a purely compositional language and that the language which supports systematicity can include some non-compositional aspects. Thus, our theory supports the generational bottleneck imposed by IL as being necessary for the emergence of structured natural language. We do, however, differ from prior models of IL (15, 16) in finding that the structure of this language does not need to be purely compositional to achieve the same degree of generalization. This is more consistent with what is observed in natural language where memorizing some non-compositional language does not hinder a human's ability to generalize. This is particularly salient in the use of idioms, which can be treated grammatically as nouns or verbs for example (forming parts of speech known as noun phrases and verb phrases respectively). In this case, without considering idioms as memorized strings, the distinction between lexicon and syntax does not hold. Moreover, the wording of idioms tends not to follow the usual rules of grammar (17). Examples exist in multiple languages, such as "Chengyu" in Chinese culture (18): phrases which remain in language in spite of their relative difficulty to remember, due to the historical significance of their origin (often fables or poems) and broad applicability (18). Finally, this also demonstrates the benefit of our mathematical taxonomy of systematicity as it allows us to make a distinction between compositional data and systematic network mappings which often has been conflated. We show that systematicity is a broader concept than leveraging compositional features.

From a neuroscience perspective we have demonstrated an important fact: that a detailed and large semantic system pushes the network towards the use of compositional language. It is plausible that a region such as the Anterior Temporal Lobe (ATL) in the human brain could serve as such a rich semantic representation (19–21). This is because the ATL is the central connection in a hub-and-spoke style model of semantics whereby multiple modality-specific representations converge on an amodal representation (22, 23). The ATL then connects bi-directionally to the surrounding regions which include those responsible for language comprehension and production, such as Wernicke's area (20, 24) and Broca's area (25, 26). Indeed, this finding is consistent with neuro-imaging and medical studies (27, 28) which consistently point towards the ATL as being damaged by semantic dementia which impairs language production. Importantly, our model is also consistent with the finding that the large semantic system would still be connected to highly specialized (functional) neural modules (29, 30). Thus, it appears likely that compositional language emerges as a consequence of IL paired with specialized neural modules, for example Wernicke's area specializing to the syntax of language (31) which are all connected to the rich semantic system of the ATL.

Our model also displays the complex interplay between evolution and experience in the emergence of functional neural modules. While the architectural modularity which results in compositional language was designed (implemented as physical modules), and thus would need to be obtained by evolution in nature, the refinement of the language in the dense network also forms an effective module. All that would be required further is to allocate a separate module with the task of learning to correct for the remaining errors on the non-compositional output. Practically, this is identical to the hand-crafted model but is due to the generational effect changing subsequent learners' experiences. Thus, the specific implementation of the meso-scale neural representation of language in the brain (32–34) may not be wholly innate but also due to a long process of learning and forgetting. Additionally, the architectural biases in this work which can be interpreted as separating common (compositional outputs) and proper (non-compositional outputs) nouns are also consistent with experimental findings which show that words are distinguished neurally with respect to the role they play in a sentence (35). The need for the emergence of the rich semantic space has also been found in the literature on critical learning periods (36, 37) where children that are not exposed to enough semantically meaningful stimuli in the early stages of their development are unable to learn language thereafter.

Finally, from a machine learning perspective we have expanded on the learning dynamics of deep linear networks (2, 3) and the theoretical formalization of systematicity (9). Due to this we have established the potential for IL based algorithms to produce systematic neural modules. In addition, the definition of the RCR which we have introduced here, while specific to linear networks in this context, is aligned to prior strategies for obtaining systematic networks. For example, attention based models (38–40) aim to decrease the number of features being passed through the network while still being able to perform the required tasks and identify objects. This is comparable to the goal of decreasing the rank of the correlations in the dataset while maintaining the cardinality. Similarly, modular network architectures (41) which show neural modules a subset of the dataset (42), aim to find a subset which has dramatically lower rank than the full dataset, but still has a large enough cardinality to produce a reusable module. Thus, the goal in these models is never to have a module which specializes to individual datapoints, but rather simple concepts (with low rank correlation) that are frequently seen (with a high cardinality). Indeed, there is likely an extended definition to the RCR which makes use of intrinsic dimensionality (43, 44) or a similar concept to replace the notion of correlation rank.

Proof of Theorem 1.

Informal Theorem 1. Given a dataset (X, Y) in the deep linear network setting having a depth of $D \approx 1 - 2\ln(\pi_0)$ maximises the distance between the escaping time of the modes, making IL as efficient as possible.

Theorem 1. *Given a dataset (X, Y) in the deep linear network setting then the depth which minimises the time derivative of a mode α by solving $\tau \frac{d}{dD}\frac{d}{dt}\pi_\alpha = 0$ is $D = 1 - 2\ln(\pi_0)$.*

The general dynamics for a deep linear neural network has been derived in (3) and follows the exact same strategy as derivation for the two-layer case we present above and focus on for the majority of this work. While no general solution exists for the closed-form dynamics of the mode strength over time, it is sufficient for our case to consider the time-derivative of the mode with the equation $\tau \frac{d}{dt}\pi_\alpha = (D-1)(\lambda_\alpha - \pi_\alpha\delta_\alpha)\pi_\alpha^{(2D-4)/(D-1)}$. We note that our aim here is to consider the time taken for the mode to reach its escaping time. Thus, we consider the mode at its initial strength and have the equation $\tau \frac{d}{dt}\pi_\alpha = (D-1)(\lambda_\alpha - \pi_0\delta_\alpha)\pi_0^{(2D-4)/(D-1)}$. To consider how the depth of the network affects the update to the mode around its initial value we consider $\tau \frac{d}{dD}\frac{d}{dt}\pi_\alpha$:

$$\begin{aligned}
\tau \frac{d}{dD}\frac{d}{dt}\pi_\alpha &= \frac{d}{dD}(D-1)(\lambda_\alpha - \pi_0\delta_\alpha)\pi_0^{\frac{(2D-4)}{(D-1)}} \\
&= \left[\frac{d}{dD}(D-1)\right](\lambda_\alpha - \pi_0\delta_\alpha)\pi_0^{\frac{(2D-4)}{(D-1)}} + (D-1)\left[\frac{d}{dD}(\lambda_\alpha - \pi_0\delta_\alpha)\pi_0^{\frac{(2D-4)}{(D-1)}}\right] \\
&= (\lambda_\alpha - \pi_0\delta_\alpha)\pi_0^{\frac{(2D-4)}{(D-1)}} + (D-1)(\lambda_\alpha - \pi_0\delta_\alpha)\left[\frac{d}{dD}\pi_0^{\frac{(2D-4)}{(D-1)}}\right] \\
&= (\lambda_\alpha - \pi_0\delta_\alpha)\pi_0^{\frac{(2D-4)}{(D-1)}} + (\lambda_\alpha - \pi_0\delta_\alpha)\frac{2\pi_0^{\frac{(2D-4)}{(D-1)}}\ln(\pi_0)}{(D-1)} \\
&= (\lambda_\alpha - \pi_0\delta_\alpha)\pi_0^{\frac{(2D-4)}{(D-1)}}\left[1 + \frac{2\ln(\pi_0)}{(D-1)}\right]
\end{aligned}$$

We then consider when this will be negative such that adding more layers will slow down the initial learning of the mode:

$$\begin{aligned}
\tau \frac{d}{dD}\frac{d}{dt}\pi_\alpha &< 0 \\
(\lambda_\alpha - \pi_0\delta_\alpha)\pi_0^{\frac{(2D-4)}{(D-1)}}\left[1 + \frac{2\ln(\pi_0)}{(D-1)}\right] &< 0 \\
1 + \frac{2\ln(\pi_0)}{(D-1)} &< 0 \\
D - 1 + 2\ln(\pi_0) &< 0 \\
D &< 1 - 2\ln(\pi_0)
\end{aligned}$$

For an initial mode strength of $\pi_0 = 0.001$ this results in $D \approx 15$, and for $\pi = 0.0001, D \approx 19$. Thus, for an initial mode strength there is a specific depth where the network learns the slowest at the start of learning. Consequently all modes will reach their escaping time slower, and as learning time is a function of the input-output correlation mode strengths, this has the effect of stretching the learning dynamics of the network. This means that the distance between each mode's learning strength will be maximised at this depth. The fact that increasing depth slows learning and strengths the dynamics was noted in Saxe et al. (2019) (3), however a computation of the optimal depth is new to this work.

Proof of Theorem 2.

Informal Theorem 2. Given a dataset (X, Y) in the deep linear network setting having multiple generations of learners is a **necessary** condition for guaranteed removal of only the desired modes of variation.

Theorem 2. *Given a dataset (X, Y), and assuming small random initial network parameters, a small learning rate ϵ and that removable modes have smaller singular values than maintained modes, $G > 0$ (having multiple generations of learners) is a* ***necessary*** *condition for guaranteed removal of only the desired modes of variation.*

We note that for now we make no assumptions on the dataset or singular values aside from their usual ordering. In subsequent sections we will make reference to the particular space of datasets. We now aim to show that there will always be a $G \geq 0$ for which the removable mode's hitting time will be larger than the maintained modes convergence time. To begin we need to show that the convergence time of the maintained mode is larger than the convergence time of the removable mode for all generations. This means that it is possible to find a time where the maintained mode has converged but the removable mode has not and IL is a valid algorithm for removing the removable mode. We may keep G general here as the generation does not change the proof since a removable mode can only decrease in size. Thus, we begin by determining under what conditions the maintained mode will have an earlier convergence time than the removable modes:

$$\begin{aligned}
t^*_{\alpha-1} &> t^*_\alpha \\
\frac{\tau}{2\lambda^G_{\alpha-1}}\log\left(\frac{\lambda^{G^2}_{\alpha-1} - \delta_{\alpha-1}\lambda^G_{\alpha-1}\pi_0 - \delta_{\alpha-1}\lambda^G_{\alpha-1}\rho + \delta^2_{\alpha-1}\pi_0\rho}{\delta^2_{\alpha-1}\pi_0\rho}\right) &> \frac{\tau}{2\lambda^G_\alpha}\log\left(\frac{\lambda^{G^2}_\alpha - \delta_\alpha\lambda^G_\alpha\pi_0 - \delta_\alpha\lambda^G_\alpha\rho + \delta^2_\alpha\pi_0\rho}{\delta^2_\alpha\pi_0\rho}\right)
\end{aligned}$$

We next substitute in the fact that $\pi_0 \to 0$ and $\rho \to 0$. However, we need to consider first that $\lambda^G_{\alpha-1}$ may be small (especially when IL is being applied). The above inequality will only hold when the log on the left of the inequality is positive. Thus, we first consider when the argument to the log is greater than 1:

$$\frac{\lambda^{G^2}_{\alpha-1} - \delta_{\alpha-1}\lambda^G_{\alpha-1}\pi_0 - \delta_{\alpha-1}\lambda^G_{\alpha-1}\rho + \delta^2_{\alpha-1}\pi_0\rho}{\delta^2_{\alpha-1}\pi_0\rho} > 1$$

$$\frac{\lambda^{G^2}_{\alpha-1} - \delta_{\alpha-1}\lambda^G_{\alpha-1}\pi_0 - \delta_{\alpha-1}\lambda^G_{\alpha-1}\rho}{\delta^2_{\alpha-1}\pi_0\rho} + 1 > 1$$

$$\frac{\lambda^{G^2}_{\alpha-1} - \delta_{\alpha-1}\lambda^G_{\alpha-1}\pi_0 - \delta_{\alpha-1}\lambda^G_{\alpha-1}\rho}{\delta^2_{\alpha-1}\pi_0\rho} > 0$$

$$\lambda^G_{\alpha-1} - \delta_{\alpha-1}\pi_0 - \delta_{\alpha-1}\rho > 0$$

$$\frac{\lambda^G_{\alpha-1}}{\delta_{\alpha-1}} > \pi_0 + \rho$$

Thus, for the log to be positive the final value of the mode must be larger than $\pi_0 + \rho$. This is a very easy constraint to meet and any mode with a final value less than $\pi_0 + \rho$ will have no real bearing on the output language. Additionally, in the limits of $\pi_0 \to 0$ and $\rho \to 0$ this just means that the final mode must be greater than 0 which is true by definition. We are free to substitute in $\pi_0 \to 0$ and $\rho \to 0$ to the original expression and it simplifies to the following where $c = \frac{1}{\pi_0\rho}$.

$$\frac{1}{2\lambda^G_{\alpha-1}} \log\left(c\frac{\lambda^{G^2}_{\alpha-1}}{\delta^2_{\alpha-1}}\right) > \frac{1}{2\lambda^G_{\alpha}} \log\left(c\frac{\lambda^{G^2}_{\alpha}}{\delta^2_{\alpha}}\right)$$

$$\frac{1}{\lambda^G_{\alpha-1}} \log\left(\sqrt{c}\frac{\lambda^{G}_{\alpha-1}}{\delta_{\alpha-1}}\right) > \frac{1}{\lambda^G_{\alpha}} \log\left(\sqrt{c}\frac{\lambda^{G}_{\alpha}}{\delta_{\alpha}}\right)$$

What this demonstrates is that, with small initial mode values and a high precision, the convergence time is inversely related to the size of the input-output correlation of the mode and proportional to the log of the final value of the mode itself. In essence, how long it takes learning to converge depends on how quickly learning happens and how much there is to learn. Noting that we can set c inside of the log and know that it is a very large value ($c \to \infty$) we can put the expression in a regime where the log derivative is near 0 and we can treat the two log expressions as constant and equal. We note that this is only valid where the log is positive which we have demonstrated is the case. How large we need to set c in practice depends entirely on the relative scale of $\lambda^G_{\alpha-1}/\delta_{\alpha-1}$ and $\lambda^G_{\alpha}/\delta_{\alpha}$. If $\lambda^G_{\alpha-1}/\delta_{\alpha-1}$ and $\lambda^G_{\alpha}/\delta_{\alpha}$ are of roughly the same magnitude then it may not even be necessary to set a large c for the inequality to hold, but this then depends on the relative scale of $\lambda^G_{\alpha-1}$ and λ^G_{α} in isolation too. What we are accounting for here is the case where a slower mode also has a much smaller final value, in which case it may still converge as quickly as the faster learning mode. The way to ensure these modes maintain their ordering is to set a smaller initial parameters and a smaller precision. This works because the dynamics have the sigmoidal training curve where overcoming the initial saddle point takes long and then there is a stage-like transition once the mode begins being learned. This simplifies the expression further to:

$$\frac{1}{\lambda^G_{\alpha-1}} \gtrapprox \frac{1}{\lambda^G_{\alpha}}$$

This is true by definition and so it is appropriate to apply IL for all generations. We will now consider the relationship between the removable modes hitting time and the maintained modes convergence time.

$$\hat{t}_{\alpha-1} > t^*_{\alpha}$$

$$\frac{\tau}{2\lambda^G_{\alpha-1}} \log\left(\frac{\lambda^G_{\alpha-1}\rho - \delta_{\alpha-1}\pi_0\rho}{\lambda^G_{\alpha-1}\pi_0 - \delta_{\alpha-1}\pi_0\rho}\right) > \frac{\tau}{2\lambda^G_{\alpha}} \log\left(\frac{\lambda^{G^2}_{\alpha} - \delta_{\alpha}\lambda^G_{\alpha}\pi_0 - \delta_{\alpha}\lambda^G_{\alpha}\rho + \delta^2_{\alpha}\pi_0\rho}{\delta^2_{\alpha}\pi_0\rho}\right)$$

Once again, applying the usual limits of $\pi_0 \to 0$ and $\rho \to 0$ simplifies the expression to:

$$\frac{1}{\lambda^G_{\alpha-1}} \log\left(\frac{\lambda^G_{\alpha-1}\rho}{\lambda^G_{\alpha-1}\pi_0}\right) > \frac{1}{\lambda^G_{\alpha}} \log\left(c\frac{\lambda^{G^2}_{\alpha}}{\delta^2_{\alpha}}\right)$$

$$\frac{1}{\lambda^G_{\alpha-1}} \log\left(\frac{\rho}{\pi_0}\right) > \frac{2}{\lambda^G_{\alpha}} \log\left(\sqrt{c}\frac{\lambda^{G}_{\alpha}}{\delta_{\alpha}}\right)$$

$$\frac{1}{\lambda^G_{\alpha-1}} \log\left(\frac{\rho}{\pi_0}\right) > \frac{2}{\lambda^G_{\alpha}} \log\left(\sqrt{c}\frac{\lambda^{G}_{\alpha}}{\delta_{\alpha}}\right)$$

$$\frac{1}{\lambda^G_{\alpha-1}} \log\left(\rho^2 c\right) > \frac{2}{\lambda^G_{\alpha}} \log\left(\sqrt{c}\frac{\lambda^{G}_{\alpha}}{\delta_{\alpha}}\right)$$

$$
\begin{aligned}
\frac{2}{\lambda^G_{\alpha-1}}\log\left(\rho\sqrt{c}\right) >& \frac{2}{\lambda^G_\alpha}\log\left(\sqrt{c}\frac{\lambda^G_\alpha}{\delta_\alpha}\right)\\
\frac{1}{\lambda^G_{\alpha-1}}\log\left(\rho\sqrt{c}\right) >& \frac{1}{\lambda^G_\alpha}\log\left(\frac{\lambda^G_\alpha}{\delta_\alpha}\sqrt{c}\right)\\
\frac{1}{\lambda^G_{\alpha-1}}\log\left(\frac{\rho}{\sqrt{\rho\pi_0}}\right) >& \frac{1}{\lambda^G_\alpha}\log\left(\frac{\lambda^G_\alpha}{\delta_\alpha}\frac{1}{\sqrt{\rho\pi_0}}\right)\\
\frac{1}{\lambda^G_{\alpha-1}}\log\left(\frac{\sqrt{\rho}}{\sqrt{\pi_0}}\right) >& \frac{1}{\lambda^G_\alpha}\log\left(\frac{\lambda^G_\alpha}{\delta_\alpha}\frac{1}{\sqrt{\rho\pi_0}}\right)
\end{aligned}
$$

This expression is not true in general. For example if we set: $\lambda^G_{\alpha-1} = 1$, $\lambda^G_\alpha = \sqrt{2}$ and $\delta_\alpha = 1$ then we obtain:

$$
\begin{aligned}
\frac{1}{2}\log\left(\frac{\rho}{\pi_0}\right) >& \frac{1}{2\sqrt{2}}\log\left(\frac{2}{\rho\pi_0}\right)\\
\log\left(\frac{\rho}{\pi_0}\right) >& \frac{1}{\sqrt{2}}\log\left(\frac{2}{\rho\pi_0}\right)
\end{aligned}
$$

However $\rho \to 0$ and the left side of the expression is tending towards 0 while the right side is tending towards ∞. Thus, this is a contradiction. However, if we use the fact that $\lambda^G_{\alpha-1} \to 0$ as would be the case when the IL algorithm is applied, then regardless of the other dataset statistics the expression simplifies to:

$$
\begin{aligned}
\lim_{G\to\infty}\frac{1}{\lambda^G_{\alpha-1}}\log\left(\frac{\rho}{\pi_0}\right) >& \lim_{G\to\infty}\frac{2}{\lambda^G_\alpha}\log\left(\sqrt{c}\frac{\lambda^G_\alpha}{\delta_\alpha}\right)\\
\frac{1}{\lambda^\infty_{\alpha-1}}\log\left(\frac{\rho}{\pi_0}\right) >& \frac{2}{\lambda^G_\alpha}\log\left(\sqrt{c}\frac{\lambda^G_\alpha}{\delta_\alpha}\right)\\
\infty >& \frac{2}{\lambda^G_\alpha}\log\left(\sqrt{c}\frac{\lambda^G_\alpha}{\delta_\alpha}\right)
\end{aligned}
$$

This is true by definition. In practice all that is required is to find some G for which the expression holds by a sufficient decrease in $\lambda^G_{\alpha-1}$. Thus, we have shown that it is always possible to reach a point where a removable mode's hitting time is higher than the maintained modes convergence time. This is not guaranteed to be the case for the first generation and may require multiple generations, which have shown to always be a viable strategy. As a consequence it is always possible to remove a removable mode while maintaining the maintained mode.

Proof of Observation 5.

Observation 1. *For all points in the space of datasets: $n_x, n_y, k_x, k_y, r \in \mathbb{Z}^+$ the input-output correlation matrix Σ^{yx} singular values will be ordered as: $\lambda_1 > \lambda_2 > \lambda_3$.*

Firstly we prove that $\lambda_1 > \lambda_2$:

$$
\begin{aligned}
\lambda_1 >& \lambda_2\\
\left(\frac{(k_x r^2 + 2^{n_x})(k_y r^2 + 2^{n_x})}{2^{2n_x}}\right)^{\frac{1}{2}} >& \left(\frac{(k_x r^2 + 2^{n_x})(k_y r^2)}{2^{2n_x}}\right)^{\frac{1}{2}}\\
\left((k_x r^2 + 2^{n_x})(k_y r^2 + 2^{n_x})\right) >& \left((k_x r^2 + 2^{n_x})(k_y r^2)\right)\\
k_y r^2 + 2^{n_x} >& k_y r^2\\
2^{n_x} >& 0
\end{aligned}
$$

$2^{n_x} > 0$ is true by definition since $n_x \in \mathbb{Z}^+$ and, thus, $\lambda_1 > \lambda_2$ for all points in our space of datasets. Now we prove that $\lambda_2 > \lambda_3$:

$$
\begin{aligned}
\lambda_2 >& \lambda_3\\
\left(\frac{(k_x r^2 + 2^{n_x})(k_y r^2)}{2^{2n_x}}\right)^{\frac{1}{2}} >& \left(\frac{k_x k_y r^4}{2^{2n_x}}\right)^{\frac{1}{2}}\\
(k_x r^2 + 2^{n_x})(k_y r^2) >& k_x k_y r^4\\
k_x k_y r^4 + 2^{n_x} k_y r^2 >& k_x k_y r^4\\
2^{n_x} k_y r^2 >& 0
\end{aligned}
$$

$2^{n_x} k_y r^2 > 0$ is true by definition since $n_x, k_y, r \in \mathbb{Z}^+$ and, thus, $\lambda_2 > \lambda_3$ for all points in our space of datasets. Thus, using the transitivity of inequality: $\lambda_1 > \lambda_2 > \lambda_3$ for all points in the space of datasets.

Proof of Observation 6.

Observation 2. *For all points in the space of datasets: $n_x, n_y, k_x, k_y, r \in \mathbb{Z}^+$ IL identifies exploitable low-rank substructure in the input-output correlation (Σ^{yx}) and is output systematic.*

We begin by noting that the λ_1 modes contain all of the information for mapping between compositional input and compositional output features - which are sufficient to identify each datapoint. Thus, we will use λ_1 as the maintained modes and λ_2 and λ_3 as the removable modes. Then, due to Observation 1 we know that the maintained modes will learn faster than the removable modes. This fact with Theorem 2 then means that IL is a valid algorithm for removing the removable modes and that there is some $G > 0$ for which it is guaranteed that the removable modes will be removed. Thus, $\pi_2^G \to 0$ and $\pi_3^G \to 0$ while $\pi_1^G = \pi_1^0$. The rank then of the new input-output mapping is just the multiplicity of $|\pi_1^G|$ compared to the sum of multiplicities of all modes in the original dataset $|\pi_1^0| + |\pi_2^0| + |\pi_3^0|$. We show that the new rank is lower than the original dataset rank:

$$\begin{aligned} |\pi_1^0| + |\pi_2^0| + |\pi_3^0| &> |\pi_1^G| \\ |\pi_1^0| + |\pi_2^0| + |\pi_3^0| &> |\pi_1^0| \\ |\pi_2^0| + |\pi_3^0| &> 0 \end{aligned}$$

This is true by definition and so the rank has been decreased. We note, however, that since the mode π_1^G provides a unique set of features for all datapoints in the original dataset (even though there are naturally now errors in some of these features) the cardinality of the dataset has remained the same. Thus, the network trained on this new mapping will have a lower RCR for the input-output correlation than a network which learns the full mapping on the original dataset and as a consequence is output systematic by Definition 3.2 in the main text.

Proof of Observation 7.

Observation 3. *For $n_x, n_y, k_x, k_y, r \in \mathbb{Z}^+$, as $n_x \to \infty$ the association between non-compositional input and compositional output is removed: $X_\Gamma Y_\Omega^G$-Norm $\to 0$.*

To begin we will consider the equation for the dynamics of the $X_\Gamma Y_\Omega^G$-Norm

$$\begin{aligned} X_\Gamma Y_\Omega^G\text{-Norm} &= \left(\frac{2^{n_x} n_y k_x r^2 \pi_1^2(t)}{(k_x r^2 + 2^{n_x})(k_y r^2 + 2^{n_x})} \right)^{\frac{1}{2}} \\ &= \left(\frac{n_y k_x r^2 \pi_1^2(t)}{2^{n_x}(\frac{k_x r^2}{2^{n_x}} + 1)(\frac{k_y r^2}{2^{n_x}} + 1)} \right)^{\frac{1}{2}} \end{aligned}$$

If we now take $n_x \to \infty$ the expression simplifies as follows:

$$\begin{aligned} X_\Gamma Y_\Omega^G\text{-Norm} &= \left(\frac{n_y k_x r^2 \pi_1^2(t)}{2^{n_x}(\frac{k_x r^2}{2^{n_x}} + 1)(\frac{k_y r^2}{2^{n_x}} + 1)} \right)^{\frac{1}{2}} \\ &= \left(\frac{1}{2^{n_x}} (n_y k_x r^2 \pi_1^2(t)) \right)^{\frac{1}{2}} \\ &= 0 \end{aligned}$$

Proof of Proposition 1.

Proposition 1. *Given a dataset (X, Y) removing an input or output feature cannot increase the first singular value of the input-output correlation matrix Σ^{yx}.*

To begin we prove a generalization of the Cauchy Interlacing Theorem for non-square matrices which closely follows the strategy of Thompson (1972) (45). The aim of this is to show that if an input or output feature is removed from a dataset that the first singular value will decrease.

For any matrix M lets denote the submatrix of rows $i_1, ..., i_p$ and columns $j_1, ..., j_q$ as $M[i_1, ..., i_p | j_1, ..., j_q]$. To ease notation let $\mu = \{i_1, ..., i_p\}$ and $\nu = \{j_1, ..., j_q\}$ for $p < m$ and $q < n$. Then let $B = \Sigma_{yx}[\mu|\nu]$ a submatrix of the correlation matrix $\Sigma_{yx} \in \mathbb{R}^{m \times n}$. If $U = \mathbb{I}_m$ and $V = \mathbb{I}_n$ (the identity matrices of m and n dimensions) then B can also be written as $B = U[\mu|1, ..., m]\Sigma_{yx} V[1, .., n|\nu]$. Thus:

$$\begin{aligned} BB^T &= U[\mu|1, ..., m]\Sigma_{yx} V[1, .., n|\nu] V^T[\nu|1, .., n] \Sigma_{yx}^T U^T[1, ..., m|\mu] \\ &= U[\mu|1, ..., m] AA^T U^T[1, ..., m|\mu] \end{aligned}$$

where $A = \Sigma_{yx} V[1,..,n|\nu] \in \mathbb{R}^{m\times q}$ with ordered, non-zero singular values of $\alpha_1, \alpha_2, ..., \alpha_{\min(m,q)}$. Thus BB^T is a principal p-square submatrix of the m-square symmetric matrix AA^T. Since BB^T is guaranteed to be symmetric we know it will be diagonalizable. Thus, there exists an orthonormal basis of eigenvectors $\{b_1, ..., b_{\min(p,q)}\}$ corresponding to eigenvalues $\{\beta_1^2, ..., \beta^2_{\min(p,q)}\}$. We define $G_j = \text{span}[b_1, ..., b_j]$ (the span of the first j eigenvectors of BB^T) for $j \leq \min(p, q)$ and $S_j = \text{span}[a_j, ..., a_{\min(m,q)}]$ (the span of the last $\min(m, q) - j + 1$ eigenvectors of AA^T). We also define the subspace:

$$H_j = \left\{U^T[1, ..., m|\mu]g, g \in G_j\right\}$$

There exits a unit length vector $\tilde{z} = U^T[1, ..., m|\mu]z$ for $z \in G_j$ which lies in $H_j \cap S_j$, as if there is not then the dimension of $H_j \cap S_j$ would be $j + \min(m, q) - j + 1$ which is impossible in $\mathbb{R}^{\min(m,q)}$. Additionally:

$$z = \sum_{i=1}^{j} r_i b_i \qquad \text{and} \qquad \langle z, z\rangle = z^T z = \sum_{i=1}^{j} r_i^2 = 1$$

(since the eigenvectors of symmetric matrices are orthogonal). We begin by considering the Rayleigh–Ritz quotient for BB^T and another unit length vector $v \in G_j$:

$$R_{BB^T}(v) = \frac{\langle BB^T v, v\rangle}{\langle v, v\rangle} = v^T BB^T v = \sum_{i=1}^{\min(p,q)} \beta_i^2 r_i^2$$

Here r_i^2 acts as a weighting on each eigenvalue for how much it contributes to the sum. Thus to minimize the quotient we place all of the weighting on the lowest eigenvalue β_j^2 by picking v such that $r_i = 0$ for $i \in \{1, ..., j-1\}$ and $r_j = 1$. This result is known as the Min-Max Theorem. A similar result can be shown where the j-th eigenvalue also results from maximizing the quotient over the span of the bottom eigenvector beginning with the j-th eigenvector. Thus:

$$\begin{aligned}
\beta_j^2 &= \min_{v\in G_j;||v||=1} R_{BB^T}(v) \text{ by the Min-Max Theorem} \\
&= \min_{v\in G_j;||v||=1} \langle BB^T v, v\rangle \\
&\leq \langle BB^T z, z\rangle \text{ since } z \in G_j \text{ does not necessarily minimize the quotient} \\
&= \langle U[\mu|1, ..., m]AA^T U^T[1, ..., m|\mu]z, z\rangle \\
&= \langle AA^T \left(U^T[1, ..., m|\mu]z\right), U^T[1, ..., m|\mu]z\rangle \text{ by the linearity of the inner product} \\
&= \langle AA^T \tilde{z}, \tilde{z}\rangle \\
&\leq \max_{\tilde{v}\in S_j;||v||=1} \langle AA^T \tilde{v}, \tilde{v}\rangle \text{ since } \tilde{z} \in S_j \text{ does not necessarily maximize the quotient like } \tilde{v} \\
&= \max_{\tilde{v}\in S_j;||v||=1} R_{AA^T}(\tilde{v}) \\
&= \alpha_j^2 \text{ by the Min-Max Theorem}
\end{aligned}$$

Thus $\beta_j \leq \alpha_j$. Similarly, $A^T A = V^T[\nu|1, .., n]\Sigma_{yx}^T \Sigma_{yx} V[1, .., n|\nu]$ with eigenvalues of $\alpha_1^2, \alpha_2^2, ..., \alpha^2_{\min(m,q)}$. Thus, $A^T A$ is a principal q-square submatrix of the n-square symmetric matrix $V^T \Sigma_{yx}^T \Sigma_{yx} V$. Since $A^T A$ is guaranteed to be symmetric we know it will be diagonalizable. Thus, there exists an orthonormal basis of eigenvectors $\{a_1, ..., a_{\min(m,q)}\}$ corresponding to eigenvalues $\{\alpha_1^2, ..., \alpha^2_{\min(m,q)}\}$. We overload the notation and define $G_j = \text{span}[a_1, ..., a_j]$ (the span of the first j eigenvectors of $A^T A$) for $j \leq \min(m, q)$ and $S_j = \text{span}[\sigma_j, ..., \sigma_{\min(m,n)}]$ (the span of the last $\min(m, n) - j + 1$ eigenvectors of $\Sigma_{yx}^T \Sigma_{yx}$). We also define the subspace:

$$H_j = \{V[1, .., n|\nu]g, g \in G_j\}$$

There exits a unit length vector $\tilde{z} = V[1, .., n|\nu]z$ for $z \in G_j$ which lies in $H_j \cap S_j$, as if there is not then the dimension of $H_j \cap S_j$ would be $j + \min(m, n) - j + 1$ which is impossible in $\mathbb{R}^{\min(m,n)}$. Additionally:

$$z = \sum_{i=1}^{j} r_i a_i \qquad \text{and} \qquad \langle z, z\rangle = z^T z = \sum_{i=1}^{j} r_i^2 = 1$$

(since the eigenvectors of symmetric matrices are orthogonal). We begin by considering the Rayleigh–Ritz quotient for $A^T A$ and another unit length vector $v \in G_j$:

$$R_{A^T A}(v) = \frac{\langle A^T A v, v\rangle}{\langle v, v\rangle} = v^T A^T A v = \sum_{i=1}^{\min(m,q)} \alpha_i^2 r_i^2$$

Here r_i^2 acts as a weighting on each eigenvalue for how much it contributes to the sum. Thus to minimize the quotient we place all of the weighting on the lowest eigenvalue α_j^2 by picking v such that $r_i = 0$ for $i \in \{1, ..., j-1\}$ and $r_j = 1$:

$$\begin{aligned}
\alpha_j^2 &= \min_{v \in G_j; ||v||=1} R_{A^TA}(v) \text{ by the Min-Max Theorem} \\
&= \min_{v \in G_j; ||v||=1} \langle A^T A v, v \rangle \\
&\leq \langle A^T A z, z \rangle \text{ since } z \in G_j \text{ does not necessarily minimize the quotient} \\
&= \langle V^T[\nu|1, .., n] \Sigma_{yx}^T \Sigma_{yx} V[1, .., n|\nu] z, z \rangle \\
&= \langle \Sigma_{yx}^T \Sigma_{yx} \left(V[1, .., n|\nu] z \right), V[1, .., n|\nu] z \rangle \text{ by the linearity of the inner product} \\
&= \langle \Sigma_{yx}^T \Sigma_{yx} \tilde{z}, \tilde{z} \rangle \\
&\leq \max_{\tilde{v} \in S_j; ||v||=1} \langle \Sigma_{yx}^T \Sigma_{yx} \tilde{v}, \tilde{v} \rangle \text{ since } \tilde{z} \in S_j \text{ does not necessarily maximize the quotient like } \tilde{v} \\
&= \max_{\tilde{v} \in S_j; ||v||=1} R_{\Sigma_{yx}^T \Sigma_{yx}}(\tilde{v}) \\
&= \sigma_j^2 \text{ by the Min-Max Theorem}
\end{aligned}$$

Thus $\alpha_j \leq \sigma_j$. Putting both inequalities together we obtain the result: $\beta_j \leq \alpha_j \leq \sigma_j$ with the particularly important case of $\beta_1 \leq \sigma_1$. This means that a shared pathway will always be learned faster than a pathway which considers only a subset of the input features or output labels (which input features is determined by μ and which output labels is determined by ν).

Proof of Observation 0.1

Observation 0.1. *For all points in the space of datasets: $n_x, n_y, k_x, k_y, r \in \mathbb{Z}^+$ with the dense network architecture: $X_\Omega Y_\Omega^G$-Norm will reach its hitting time in the same time course as $X_\Omega Y_\Gamma^G$-Norm and $X_\Gamma Y_\Gamma^G$-Norm $\forall\ G > 0$.*

We note that for the Frobenius norms the hitting time is the minimum of the hitting times of all modes which contribute to that norm. Thus for the $X_\Omega Y_\Omega^G$-Norm of Equation 14 it only has a single contributing mode of $\pi_1(t)$ with a hitting time of:

$$\hat{t}_1 = \frac{\tau}{2\lambda_1^G} \log\left(\frac{\lambda_1^G \rho - \delta_1 \pi_0 \rho}{\lambda_1^G \pi_0 - \delta_1 \pi_0 \rho} \right)$$

For the $X_\Omega Y_\Gamma^G$-Norm in Equation 16 it receives contributions from the $\pi_1(t)$ and $\pi_2(t)$ modes. For the $X_\Gamma Y_\Gamma^G$-Norm in Equation 17 it receives contributions from all three modes $\pi_1(t), \pi_2(t)$ and $\pi_3(t)$. Thus, we need to consider which of the three modes has the minimal hitting time, where $\pi_2(t)$ and $\pi_3(t)$ have hitting times of:

$$\hat{t}_2 = \frac{\tau}{2\lambda_2^G} \log\left(\frac{\lambda_2^G \rho - \delta_1 \pi_0 \rho}{\lambda_2^G \pi_0 - \delta_1 \pi_0 \rho} \right);\ \hat{t}_3 = \frac{\tau}{2\lambda_3^G} \log\left(\frac{\lambda_3^G \rho - \delta_2 \pi_0 \rho}{\lambda_3^G \pi_0 - \delta_2 \pi_0 \rho} \right)$$

Noting that $\pi_0 \to 0$ and $\rho \to 0$ these hitting times simplify to:

$$\hat{t}_1 = \frac{\tau}{2\lambda_1^G} \log\left(\frac{\rho}{\pi_0} \right);\ \hat{t}_2 = \frac{\tau}{2\lambda_2^G} \log\left(\frac{\rho}{\pi_0} \right);\ \hat{t}_3 = \frac{\tau}{2\lambda_3^G} \log\left(\frac{\rho}{\pi_0} \right)$$

From Observation 1 we know that the singular values will be ordered as: $\lambda_1 > \lambda_2 > \lambda_3$. Consequently we know that:

$$\frac{\tau}{2\lambda_3^G} \log\left(\frac{\rho}{\pi_0} \right) > \frac{\tau}{2\lambda_2^G} \log\left(\frac{\rho}{\pi_0} \right) > \frac{\tau}{2\lambda_1^G} \log\left(\frac{\rho}{\pi_0} \right)$$
$$\hat{t}_3 > \hat{t}_2 > \hat{t}_1$$

Thus, the hitting time for all three norms: $X_\Omega Y_\Omega^G$-Norm, $X_\Omega Y_\Gamma^G$-Norm and $X_\Gamma Y_\Gamma^G$-Norm will depend on the hitting time of $\hat{t}_1$. As a result all norms have the same hitting time and will begin learning in the same time-course.

Proof of Observation 0.2

Observation 0.2. *For all points in the space of datasets: $n_x, n_y, k_x, k_y, r \in \mathbb{Z}^+$ with the output-partitioned network architecture: $X_\Omega Y_\Omega^G$-Norm and $X_\Gamma Y_\Omega^G$-Norm will reach their convergence time before $X_\Omega Y_\Gamma^G$-Norm and $X_\Gamma Y_\Gamma^G$-Norm reach their hitting time for some $G > 0$.*

We note that for the Frobenius norms their hitting time is equal to the minimum hitting time of all modes which contribute to it and their convergence time is equal to the maximum convergence time of all the modes which contribute to it. In the split network architecture the Frobenius norms are shown in Equations 18-21. The $X_\Omega Y_\Omega^G$-Norm and $X_\Gamma Y_\Omega^G$-Norm depend only on $\pi_1(t)$ and have a convergence time of t_1^*. The $X_\Omega Y_\Gamma^G$-Norm depends on $\pi_2(t)$ and $X_\Gamma Y_\Gamma^G$-Norm depends on $\pi_2(t)$ and $\pi_3(t)$ respectively. Consequently, the hitting time of both $X_\Omega Y_\Gamma^G$-Norm and $X_\Gamma Y_\Gamma^G$-Norm is $\hat{t}_2$. For $X_\Gamma Y_\Gamma^G$-Norm this is because we

know from the proof of Observation 0.1 that $\pi_3(t)$ has a larger hitting time than $\pi_2(t)$ when $\pi_0 \to 0$ and $\rho \to 0$ for the space of datasets. From Observation 1 we know that the modes will be ordered as $\lambda_1 > \lambda_2 > \lambda_3$, which satisfies the assumption of Theorem 2. Then, due to Theorem 2 there is a generation $G > 0$ for which we are guaranteed to have $\hat{t}_2 > t_1^*$. Thus, there is some $G > 0$ for which the convergence time of $X_\Omega Y_\Omega^G$-Norm and $X_\Gamma Y_\Omega^G$-Norm is less than the hitting time of $X_\Omega Y_\Gamma^G$-Norm and $X_\Gamma Y_\Gamma^G$-Norm.

References

1. K Fukumizu, Effect of Batch Learning In Multilayer Neural Networks in *Proceedings of the 5th International Conference on Neural Information Processing.* pp. 67–70 (1998).
2. A Saxe, J McClelland, S Ganguli, Exact solutions to the nonlinear dynamics of learning in deep linear neural networks in *International Conference on Learning Representations*, eds. Y Bengio, Y LeCun. (Oral presentation., Banff, Canada), (2014) arXiv: 1312.6120v3.
3. AM Saxe, JL McClelland, S Ganguli, A mathematical theory of semantic development in deep neural networks. *Proc. Natl. Acad. Sci.* **116**, 11537–11546 (2019).
4. S Arora, N Cohen, E Hazan, On the optimization of deep networks: Implicit acceleration by overparameterization. *35th Int. Conf. on Mach. Learn. ICML 2018* **1**, 372–389 (2018) arXiv: 1802.06509 ISBN: 9781510867963.
5. A Lampinen, S Ganguli, An analytic theory of generalization dynamics and transfer learning in deep linear networks in *International Conference on Learning Representations*, ed. T Sainath. (2019) arXiv: 1809.10374.
6. P Baldi, K Hornik, Neural networks and principal component analysis: Learning from examples without local minima. *Neural Networks* **2**, 53–58 (1989).
7. M Huh, et al., The low-rank simplicity bias in deep networks. *arXiv preprint arXiv:2103.10427* (2021).
8. A Atanasov, B Bordelon, C Pehlevan, Neural networks as kernel learners: The silent alignment effect in *International Conference on Learning Representations.* (2021).
9. D Jarvis, R Klein, B Rosman, AM Saxe, On the specialization of neural modules in *The Eleventh International Conference on Learning Representations.* (2023).
10. H Goodglass, *Understanding aphasia.* (Academic Press), (1993).
11. G Miceli, MC Silveri, G Villa, A Caramazza, On the basis for the agrammatic's difficulty in producing main verbs. *Cortex* **20**, 207–220 (1984).
12. LB Zingeser, RS Berndt, Grammatical class and context effects in a case of pure anomia: Implications for models of language production. *Cogn. Neuropsychol.* **5**, 473–516 (1988).
13. LB Zingeser, RS Berndt, Retrieval of nouns and verbs in agrammatism and anomia. *Brain language* **39**, 14–32 (1990).
14. J Druks, Verbs and nouns—a review of the literature. *J. Neurolinguistics* **15**, 289–315 (2002).
15. ML Kalish, TL Griffiths, S Lewandowsky, Iterated learning: Intergenerational knowledge transmission reveals inductive biases. *Psychon. Bull. & Rev.* **14**, 288–294 (2007).
16. S Kirby, Spontaneous evolution of linguistic structure-an iterated learning model of the emergence of regularity and irregularity. *IEEE Transactions on Evol. Comput.* **5**, 102–110 (2001).
17. R Jackendoff, S Pinker, The nature of the language faculty and its implications for evolution of language (reply to fitch, hauser, and chomsky). *Cognition* **97**, 211–225 (2005).
18. A Tien, Compositionality of chinese idioms: the issues, the semantic approach and a case study. *Appl. Linguist. Rev.* **7**, 149–180 (2016).
19. JR Hodges, N Graham, K Patterson, Charting the progression in semantic dementia: Implications for the organisation of semantic memory. *Memory* **3**, 463–495 (1995).
20. E Jefferies, MA Lambon Ralph, Semantic impairment in stroke aphasia versus semantic dementia: a case-series comparison. *Brain* **129**, 2132–2147 (2006).
21. ML Ralph, C Lowe, TT Rogers, , et al., Neural basis of category-specific semantic deficits for living things: evidence from semantic dementia, hsve and a neural network model. *Brain* **130**, 1127–1137 (2007).
22. S Bozeat, et al., A duck with four legs: Investigating the structure of conceptual knowledge using picture drawing in semantic dementia. *Cogn. neuropsychology* **20**, 27–47 (2003).
23. RL Jackson, TT Rogers, MA Lambon Ralph, Reverse-engineering the cortical architecture for controlled semantic cognition. *Nat. human behaviour* **5**, 774–786 (2021).
24. A Ardila, B Bernal, M Rosselli, The role of wernicke's area in language comprehension. *Psychol. & Neurosci.* **9**, 340 (2016).
25. M Musso, et al., Broca's area and the language instinct. *Nat. neuroscience* **6**, 774–781 (2003).
26. M Tettamanti, D Weniger, Broca's area: a supramodal hierarchical processor? *Cortex* **42**, 491–494 (2006).
27. JT Devlin, et al., Susceptibility-induced loss of signal: comparing pet and fmri on a semantic task. *Neuroimage* **11**, 589–600 (2000).
28. M Visser, E Jefferies, M Lambon Ralph, Semantic processing in the anterior temporal lobes: a meta-analysis of the functional neuroimaging literature. *J. cognitive neuroscience* **22**, 1083–1094 (2010).
29. SE Petersen, PT Fox, MI Posner, M Mintun, ME Raichle, Positron emission tomographic studies of the cortical anatomy of single-word processing. *Nature* **331**, 585–589 (1988).
30. M Coltheart, Modularity and cognition. *Trends cognitive sciences* **3**, 115–120 (1999).

31. D Saur, et al., Ventral and dorsal pathways for language. *Proc. national academy Sci.* **105**, 18035–18040 (2008).
32. JI Flombaum, LR Santos, MD Hauser, Neuroecology and psychological modularity. *Trends Cogn. Sci.* **6**, 106–108 (2002).
33. BJ Scholl, Neural constraints on cognitive modularity? *Behav. Brain Sci.* **20**, 575–576 (1997).
34. L Cosmides, J Tooby, *Origins of domain specificity: The evolution of functional organization.* (na), (1994).
35. RC Martin, Language processing: functional organization and neuroanatomical basis. *Annu. review psychology* **54**, 55–89 (2003).
36. CE Snow, M Hoefnagel-Höhle, The critical period for language acquisition: Evidence from second language learning. *Child development* pp. 1114–1128 (1978).
37. JK Hartshorne, JB Tenenbaum, S Pinker, A critical period for second language acquisition: Evidence from 2/3 million english speakers. *Cognition* **177**, 263–277 (2018).
38. D Bahdanau, K Cho, Y Bengio, Neural machine translation by jointly learning to align and translate. *arXiv preprint arXiv:1409.0473* (2014).
39. A Vaswani, et al., Attention is all you need. *Adv. neural information processing systems* **30** (2017).
40. A Dosovitskiy, et al., An image is worth 16x16 words: Transformers for image recognition at scale. *arXiv preprint arXiv:2010.11929* (2020).
41. J Andreas, M Rohrbach, T Darrell, D Klein, Neural module networks in *Proceedings of the IEEE conference on computer vision and pattern recognition.* pp. 39–48 (2016).
42. S Mittal, Y Bengio, G Lajoie, Is a modular architecture enough? *Adv. Neural Inf. Process. Syst.* **35**, 28747–28760 (2022).
43. K Fukunaga, DR Olsen, An algorithm for finding intrinsic dimensionality of data. *IEEE Transactions on computers* **100**, 176–183 (1971).
44. A Ansuini, A Laio, JH Macke, D Zoccolan, Intrinsic dimension of data representations in deep neural networks. *Adv. Neural Inf. Process. Syst.* **32** (2019).
45. RC Thompson, Principal submatrices ix: Interlacing inequalities for singular values of submatrices. *Linear Algebr. its Appl.* **5**, 1–12 (1972).
